# General spectral characteristics of human activity and its inherent scale-free fluctuations

**DOI:** 10.1038/s41598-024-52905-8

**Published:** 2024-01-31

**Authors:** Bálint Maczák, Zoltán Gingl, Gergely Vadai

**Affiliations:** Department of Technical Informatics, University of Szeged, 6720 Szeged, Hungary

**Keywords:** Signal processing, Power law, Scale invariance

## Abstract

The scale-free nature of daily human activity has been observed in different aspects; however, the description of its spectral characteristics is incomplete. General findings are complicated by the fact that—although actigraphy is commonly used in many research areas—the activity calculation methods are not standardized; therefore, activity signals can be different. The presence of 1/*f* noise in activity or acceleration signals was mostly analysed for short time windows, and the complete spectral characteristic has only been examined in the case of certain types of them. To explore the general spectral nature of human activity in greater detail, we have performed Power Spectral Density (PSD) based examination and Detrended Fluctuation Analysis (DFA) on several-day-long, triaxial actigraphic acceleration signals of 42 healthy, free-living individuals. We generated different types of activity signals from these, using different acceleration preprocessing techniques and activity metrics. We revealed that the spectra of different types of activity signals generally follow a universal characteristic including 1/*f* noise over frequencies above the circadian rhythmicity. Moreover, we discovered that the PSD of the raw acceleration signal has the same characteristic. Our findings prove that the spectral scale-free nature is generally inherent to the motor activity of healthy, free-living humans, and is not limited to any particular activity calculation method.

## Introduction

Actigraphy is a widespread method of recording human motor activity based on collected acceleration data. Analysing such recordings is an active area of research across a range of multidisciplinary fields^1^. One of the most common applications of actigraphy is the description and analysis of the sleep quality^2^ and the circadian rhythm^3^ of the measured subjects. Actigraphy is also utilized in psychiatric examinations^4,5^, i.e., to distinguish between similar mental diseases or to recognize behavioural disorders. Besides therapeutic applications, actigraphy is also employed to study human activity patterns^6–8^. For example, to find regularities in the distribution of the resting and active periods or to examine time- and frequency domain fluctuation features of human activity, in which the power-law scaling is a recurrent motif.

**Figure 1:**
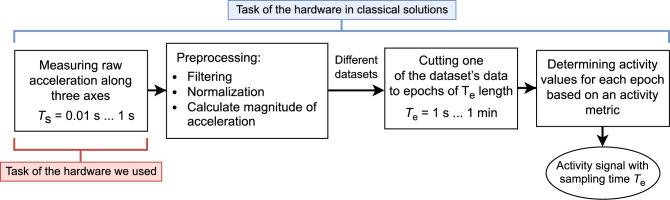
The general process of activity determination from the raw acceleration signal^1^.

Actigraphy utilizes the so-called actigraph to record the activity of the subject, which is a small, non-invasive biomedical measurement device containing a triaxial acceleration sensor, and is usually attached to the non-dominant wrist of the observed subject. Previously^1^, we have comprehensively examined and collected the diverse techniques these devices employ to derive timeslot-based activity values from the measured acceleration, this general processing scheme is presented in Fig. 1.

The classical actigraphic device generates an activity value for each consecutive, non-overlapping, equal-long timeslot of length *T*_e_ (i.e., epoch, typically 1–60 s long) based on the acceleration signal it measures (usually sampled with a rate of 1–100 Hz, along three axes), then stores the resulting activity values (i.e., the activity signal) in its memory, whose are *T*_e_ spaced in time. However, several steps of this activity determination procedure may vary between manufacturers: the raw acceleration data can be preprocessed^9–12^ in different ways (e.g., calculating the magnitude of acceleration, digital filtering, and normalization), and also numerous activity metrics^13–17^ (i.e., sets of typically non-linear operations) are available to calculate epoch-based activity values from the already preprocessed acceleration. As a consequence of these device-specific manners, human activity measures lack a standardized unit, which—often combined with incomplete methodological descriptions—makes it difficult to reproduce and compare different studies^1,11,18–20^. The clear advantage of activity calculation is that it compresses the actigraphic recordings, so it requires less resources to process them while preserving the relevant information about long-term intra- and ultradian patterns. In contrast, acceleration signals carry additional information about fast-paced, individual motions due to the higher sampling rate and by avoiding lossy compression. Nowadays, due to technological progress, actigraphs that can store the raw acceleration signal directly for several weeks are becoming increasingly common, giving the control and flexibility to calculate activity values in any desired way after the measurements.

For this reason, we have recorded raw acceleration signals of 42 healthy individuals in free-living conditions (i.e., living their normal daily routine) along three axes on their non-dominant wrist at 10 Hz in the ± 8 g measurement range for 10 days long, each. These recordings are publicly available^21^ and serve as the basis of our analysis presented in this work, too. From the raw acceleration signals, we were able to generate further non-identical types of acceleration signals based on different preprocessing methods established in the actigraphic literature. Subsequently, we could calculate numerous types of activity signals by applying different epoch-based activity metrics to the already preprocessed acceleration signals. The exact preprocessing techniques and activity metrics we utilised will be briefly summarised in the section “Materials and methods”, but are described in detail in our previous work^1^, where we also analysed the correlation between these differently produced activity signals to explore the time- and frequency-domain similarities of activity values produced by these various methods. From the correlation coefficients calculated between the different types of temporal activity signals and between their power spectral densities, an identical correlation pattern was obtained in the time- and frequency domain. Based on the correlation pattern, most activity signals showed strong similarities when calculated from identically preprocessed acceleration signals, however there may be major differences if the acceleration data is preprocessed in different ways, which is the common case for actigraphs from different manufacturers.

Our goal in this work is to examine the general patterns of human activity by analysing the spectrums of different types of actigraphic acceleration and activity signals in such a comprehensive way that was lacking in the literature so far, and which was made possible by our former^1^ extensive description and comparison of different activity determination methods. On one hand, assessing the effect of the steps of the activity calculation procedure on the observed spectral characteristic helps to understand the frequency domain relationships between differently calculated activity signals in more depth beyond the correlational similarities we had previously explored. On the other hand, we can assess the spectral correspondence between the measured acceleration and the calculated activity signals by examining how the different activity signals preserve the main spectral features of acceleration signals at lower frequencies, which is an important question, in the light of the fact that it is now feasible to conduct acceleration measurements over increasingly longer timescales. Moreover, as we shall see, the spectral characteristic—whose generality we have revealed—provides greater insight into what kind of fluctuations are present in human motor activity on a more profound level.

## Analysis of human activity patterns

In recent years, significant advances have been made in the study of temporal and spatial patterns of daily human dynamics^22,23^. In the case of human mobility, scientists have already found power-law scaling through statistical analysis^24–26^ (e.g., the spatial probability distribution of travel patterns^27^). In one of our previous works^28^ we have presented that the minute-by-minute displacement in human location data contains 1/*f*-type noise above the frequency of the daily rhythmicity which is a special form of power-law scaling in the frequency domain. Beyond human mobility, scale-free dynamics are also apparent in human activity since studies^6–8^ often identify power-law scaling in the distribution of passive periods of actigraphic recordings. However, in addition to power law, studies^29–32^ also use several other models (e.g., truncated power law, lognormal, exponential, or more complex models from the composition of the above) to explain their results, which may be influenced by the methodological differences in their analytical approach (i.e., how they separate activity signals into active/passive periods, what kind of numerical method they use to examine the distributions) in addition to the difference in their group of subjects.

Fluctuations in activity signals and their complexity have also been investigated in many cases mainly for medical and diagnostics purposes^33–36^. Such studies are typically conducted using two analytical methods: frequency-domain description through the Power Spectral Density (PSD or *S*(*f*)) and time-domain investigation based on the fluctuation function (*F*(*n*)) which is resulting from the Detrended Fluctuation Analysis (DFA).

Considering the frequency-domain-based analytical approach, fluctuations whose power spectral density *S*(*f*) is inversely proportional to the frequency are called 1/*f* noise^33,37^ (a.k.a. pink noise, or flicker noise). In other words, the PSD of 1/*f* noise follows *S*(*f*) ∝ 1/*f*^*β*^ power-law scaling, where *β* = 1. In most areas, 1/*f* type noise is associated with exponent values under more severe constraints (0.8 < *β* < 1.2^38^), while in others it is identified under less strict constraints (0.5 < *β* < 1.5^39^). Time series that exhibit such spectral properties have long-term correlations. Moreover, the integral of the PSD (i.e., the power) over equally spaced intervals on a logarithmic scale (e.g., decades) is constant for 1/*f* noise, independently of the given scale^40^, while the spectrum decays following a straight line with a slope of − *β* on log–log scales. In addition to this frequency-domain scale-free nature, what is intriguing about 1/*f* noise is that there are numerous complex systems that at first glance may appear to be very different from each other, yet they produce this type of fluctuations. Such noise has been observed in several human-made and natural phenomena, such as semiconductors^41^, urban traffic^42^, heart rate^43^, EEG signals^44^, and human activity, as explained later. To date, there is no agreed explanation or general mathematical model that implies the frequency of occurrence and universality of such noise.

Using DFA, one can compute a so-called fluctuation function *F*(*n*) of the analysed time series^45^. The algorithm splits the cumulative sum of the analysed time series into non-overlapping, equal-width boxes. The trend of each box is estimated by piecewise fitting (i.e., linear or polynomial), and then the root-mean-square deviation is calculated between the cumulative sum and the trend. The process is repeated over different box width *n* resulting in a fluctuation function *F(n)*. Similar to *S*(*f*), *F*(*n*) is mainly visualized on log–log scales. If examining 1/*f* type noise with DFA, the resulting fluctuation function should follow *F*(*n*) ∝ *n*^*α*^ power-law scaling, where *α* = 1. Both PSD and the fluctuation function describe the correlations in the analysed time series. The *α* exponent of *F*(*n*) ∝ *n*^*α*^ and the *β* exponent of *S*(*f*) ∝ 1/*f*^*β*^ are mathematically related to each other as *β* = 2 *α* – 1^46^ and both can be estimated by linear fitting over an adequate range of the log-transformed fluctuation function and power spectral density, respectively. While PSD examines the scale-free nature of time series in the frequency domain, DFA does it in the time domain.

Even though actigraphic recordings are usually several days long, the fluctuation functions of activity signals in the relevant studies are generally evaluated over timescales ranging only from a few minutes to some hours^29,47–49^, while activity signals recorded during sleep^33,34,50^ and wakefulness^51,52^ are typically analysed separately. Although these DFA-based studies identified power-law scaling, they were typically limited to a given activity type and to assess how different diseases (i.e., Alzheimer’s disease^51^, Klein-Levin disease^47^, depression^53^, bipolar disorder^54^, autism spectrum disorder^34^) or even aging^55^ break down the patterns compared to control groups without giving a detailed description of the general fluctuation patterns of human activity. Regarding frequency-domain analysis, some studies^6,56,57^ have already noted in the case of two given types of activity signals (up to 1-week-long time series, examined over their entire length) that they contain 1/*f* fluctuations above the frequency of the daily rhythmicity without giving further details on the general spectral characteristic. In conclusion, the studies found that long-term correlations and self-affinity exist in given types of human activity signals, which is an indicator of complex underlying mechanisms and regulations. However, there are many different ways of determining activity values. Considering that nonlinear operations or even simple linear ones—such as low-pass filtering—can change the spectral nature of the original stochastic signal, it is not self-evident that the observed features are indeed inherent to human activity, or that they are artefacts of the utilized activity calculation procedure.

The question emerges whether, if the activity signals have such properties, these might already be present in the acceleration signals, or they arise only as a result of the utilization of linear and nonlinear operations of activity metrics. In the literature, we found one study^46^ that investigated fluctuations in actigraphic acceleration signals instead of activity signals. Although acceleration signals can be of different types depending on the preprocessing method, they analysed the fluctuations in only two given types of acceleration signals to identify sleep–wake transitions using Welch’s method, which differs from the usual approach used for the analysis of activity signals. Because of their specific purposes, their analysed acceleration signals were considerably shorter than the activity signals commonly studied in the literature, so their spectra were limited to a narrower frequency band. Yet, they found 1/*f* noise in this frequency range of actigraphic acceleration signals recorded during wakefulness, they also confirmed their findings by DFA. This also raises the question of the extent to which their recognition can be generalised to actigraphic acceleration signals preprocessed in other ways.

In conclusion, previous studies have already partially examined the scale-free nature of human activity by analysing

- fluctuation functions of activity signals for box widths of less than 24 h typically separating into sleep and wakefulness for medical purposes^29,33,34,47–52^,
- PSDs of two given types of several-day-long activity signals over the entire frequency range^6,56,57^,
- and fluctuations of acceleration signals both with DFA and PSD (using Welch’s method) over a narrower timescale and frequency range, respectively^46^.

The preceding overview highlights several shortcomings in the literature. Firstly, the literature lacks analysing the fluctuation function of activity signals above 24 h. Moreover, the analysis over timescales of less than 24 h was focused solely on one type of activity signal, despite the existence of numerous other activity signal types. Secondly, the full-span spectral analysis has been done only for two different types of activity signals, therefore, the generality of the observed characteristics is questionable. Lastly, as the fluctuations in acceleration signals were analysed over a limited frequency band, it is a matter of question whether the acceleration signals generally follow the same full-band spectral characteristics as the activity signals do. Not to mention that there are also several ways to pre-process the acceleration signals. In this article, we aim to fill these gaps by giving the general spectral characteristic of several-day-long actigraphic recordings measured on free-living, healthy subjects, and assessing the possible differences caused by different acceleration signal processing techniques and activity metrics using PSD and DFA examination methods without separating sleep and wakefulness. Although DFA is more common in the literature, we mainly relied on PSD-based analytical approach to assess the fluctuations more accurately at low frequencies in the presence of strong periodicities. Our approach of spectral characterisation—as we will see—also captures the scale-independent nature of daily human activity, but without the heavy methodology dependence of statistical distribution analysis of active/passive periods^6–8,29–32^, while providing insight into the relationship between the acceleration signals and the activity signals calculated from them, too.

## Materials and methods

### Ways of determining human activity data

The actigraphic dataset we analysed contains 42 raw, triaxial acceleration signals of 42 free-living subjects (i.e., living their normal daily routine) measured during a 10-day observation period, each. The acceleration signals were evenly sampled with 10 Hz in the ± 8 g measurement interval with the resolution of 16 mg and were measured on the non-dominant wrist of different healthy and anonymized human subjects. The participants were instructed to minimize non-wear time, e.g., taking the device off for bathing to avoid water damage. For measuring raw acceleration recordings over an extended period, we utilized special-purpose actigraphic devices developed by us. The description of the device, the subject recruitment, and the subjects can be found in our previous article^1^. The dataset is available^21^ at Figshare under CC-BY 4.0 license.

As explained earlier, the raw acceleration signal can be preprocessed using a variety of techniques, and then an activity value can be calculated for each epoch of the preprocessed data using several different activity metrics, so the same activity metric may result in different activity signals depending on the selected preprocessing technique. In the following, we are briefly summarizing the preprocessing techniques and activity metrics we previously collected and categorised^1^, as we also used them in the current work to determine activity signals from our raw acceleration recordings. By calculating activity data afterwards the measurement of the raw acceleration data, we were able to not only examine the spectral characteristics of these differently determined activity signals but also of the underlying, variously preprocessed acceleration signals.

**Figure 2:**
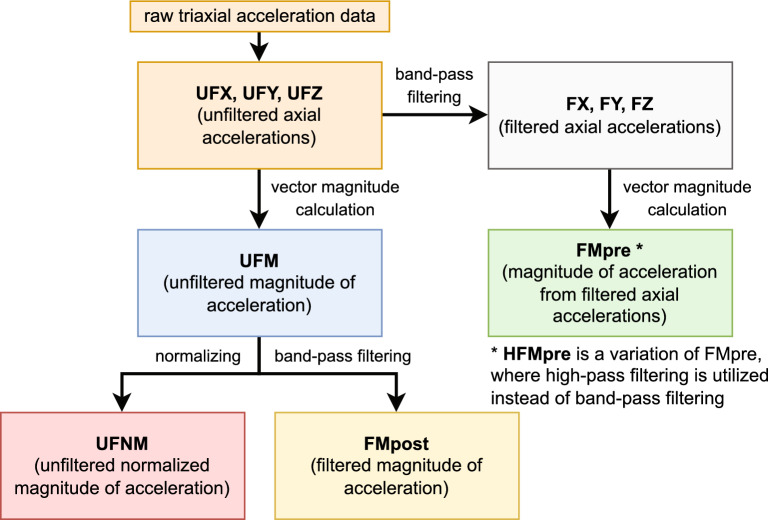
The steps of determining different types of acceleration signals from the raw triaxial acceleration data based on the different preprocessing techniques with the nomenclature that we have introduced in our previous work^1^. For band-pass filtering, a 3^rd^-order Butterworth digital filter with *f*_L_ = 0.25 Hz and *f*_H_ = 2.5 Hz was utilized. One of the activity metrics requires a variation of the FMpre acceleration signal, which is produced using a high-pass filter rather than a band-pass filter. High-pass filtering was executed using a 4^th^-order Butterworth digital filter with *f*_HP_ = 0.2 Hz.

Figure 2 summarises the different preprocessing approaches we used in the current analysis and identifies the different types of acceleration data resulting from these techniques with the nomenclature we have introduced previously^1^. From the raw acceleration of the three axes (UFX, UFY, UFZ), we can calculate the magnitude of acceleration (UFM)^12^ by taking the square root of the sum of each of the squared axial components. We can remove the effect of Earth’s gravity (*g*) from the magnitude of acceleration by determining its absolute distance from 1 g (UFNM) or by band-pass filtering it^10,12,58^ (FMpost), which removes low-frequency components including the g, and high-frequency noise, such as involuntary movements. This filtering can also be executed on a per-axis basis^59,60^ (FX, FY, FZ), and the magnitude of acceleration can be calculated from the filtered axial data, too (FMpre).

Once the acceleration data has been preprocessed in one of the previously defined ways, an epoch-based activity metric must be applied to it to calculate activity, however, multiple activity metrics are widespread in the literature because of the different working principles of available actigraphs. Table 1. summarizes the activity metrics that we have previously collected^1^ and used in the current analysis.

**Table 1:** The most common activity metrics in the literature and their brief description^1^.

Activity metric	Definition
PIM (Proportional Integration Method)^13^	It integrates the acceleration signal for a given epoch. In the following, we use the simplest numerical integration $PIM={T}_{s}\sum_{i=1}^{n}{x}_{i}$ In the formula *x*_1_, *x*_2_, …, *x*_*n* − 1_, *x*_*n*_ are the *n* acceleration values of the given epoch, and *T*_s_ is the sampling time of the acceleration signal
ZCM (Zero Crossing Method)^13^	It counts the number of times the acceleration signal crosses a *T*_ZCM_ threshold (that is equal to the standard deviation of the acceleration signal^1^ in our analysis) for each epoch
TAT (Time Above Threshold)^13^	It measures the length of time that the acceleration signal is above a *T*_TAT_ threshold (that is equal to the standard deviation of the acceleration signal^1^ in our analysis) for each epoch
MAD (Mean Amplitude Deviation)^16^	$MAD=\frac{1}{n}\sum_{i=1}^{n}\left|{r}_{i}-\overline{r }\right|$ In the formula *r*_1_, *r*_2_, …, *r*_*n* − 1_, *r*_*n*_ are the *n* magnitude of acceleration values of the given epoch, $\overline{r }$ is their arithmetic mean
ENMO (Euclidean Norm Minus One)^16,61^	$ENMO=\frac{1}{n}\sum_{i=1}^{n}{\text{max}}\left({r}_{i}-\mathrm{1,0}\right)$ In the formula *r*_1_, *r*_2_, …, *r*_*n* − 1_, *r*_*n*_ are the *n* magnitude of acceleration values of the given epoch. The values of *r* are in *g*
HFEN (High-pass Filtered Euclidean Norm)^15^	This metric requires its own, specially preprocessed acceleration signal type (previously defined as HFMpre) $HFEN=\frac{1}{n}{\sum }_{i=1}^{n}{r}_{fi}$ In the formula *r*_f1_, *r*_f2_, …, *r*_f*n* − 1_, *r*_f*n*_ are the *n* values of the HFMpre acceleration of the given epoch
AI (Activity Index)^17^	$AI=\sqrt{{\text{max}}\left(\frac{1}{3}\left(\sum_{m=1}^{3}{\sigma }_{m}^{2}-{\tilde{\sigma }}^{2}\right),0\right)}$ In the formula *m* = 1, 2, 3 corresponds to the three axes, ${\sigma }_{m}^{2}$ is the variance of the vector components along the *m*th axis of the given epoch, and ${\tilde{\sigma }}^{2}$ is the variance of the baseline noise of the total measurement data (so-called systematic noise variance)

**Figure 3:**
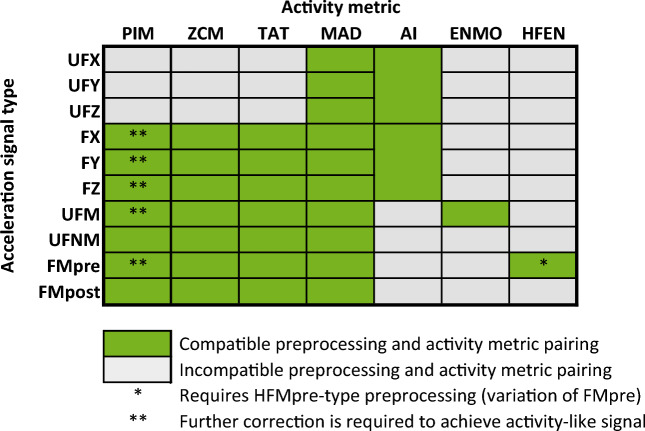
The 35 different types of activity signals whose can be generated from a single triaxial raw acceleration measurement data.

The different combinations of preprocessings and activity metrics result in different types of activity signals, however, not all activity metrics are compatible with every preprocessing technique as Fig. 3 summarises. For example, some activity metrics can only be applied to per-axis acceleration (resulting in an activity signal for each axis), while others can only be applied to the magnitude of acceleration (resulting in a single activity signal), but technical reasons may also exclude certain combinations, such as the treatment of *g* for activity metrics based on integration (for details, see^1^). For ease of interpretation, in the following, we will use the activity metric as an operation and the acceleration signal preprocessing method as its argument to denote activity signals. For example, PIM(UFNM) denotes the activity signal obtained by applying the PIM metric to the unfiltered normalized magnitude of acceleration, whereas UFNM denotes the acceleration data preprocessed in that way.

To calculate activity signals from the previously mentioned acceleration recordings, we executed the following procedure for all subjects. From a 10-day-long acceleration signal sampled with 10 Hz, an activity value was determined for each 60-s-long epoch, resulting in an activity signal with the same length but sampled at 1/60 Hz. Using the right combination of the acceleration preprocessing techniques and activity metrics (green cells of Fig. 3.), we were able to generate 35 different types of activity signals (e.g., PIM(UFNM)) and 11 different types of acceleration signals (e.g., UFNM), that all describe the 10 days of activity of the same subject but in different ways.

### Methods of examining the spectral characteristics of human activity

To describe the spectral characteristics and scaling properties of the different types of acceleration and activity signals defined earlier, we utilised the two methods prevalent in the corresponding literature: discrete Fourier transform (DFT) to assess their power spectral density *S*(*f*), and Detrended Fluctuation Analysis (DFA) with 2nd order polynomial detrending to evaluate their fluctuation function *F*(*n*). The detailed description of calculating *S*(*f*) and *F*(*n*) can be found in the supplementary material. Thus, we were able to determine 11 different types of acceleration signals and 35 different types of activity signals for each of the 42 subjects; from another point of view, we were able to calculate 42 *S*(*f*) and *F*(*n*) functions for each acceleration and activity signal type.

Subsequently, we performed ensemble averaging based on the 42 subjects to determine a general *S*(*f*) and *F*(*n*) for each acceleration and activity signal type: the 42 PSDs (and fluctuation functions) were averaged for each signal type. To do this, we reduced the noise (and resolution) of each *S*(*f*) by dividing them into logarithmically spaced bins, and after normalising the noise-reduced *S*(*f*) of the 42 subjects, we averaged them to form a single ensemble-averaged *S*(*f*). Additionally, we also calculated the ensemble-averaged total power in log-spaced frequency bins *P*(*f*) for each signal type (i.e., the integral of *S*(*f*) for each frequency bin). The detailed technical description of the ensemble-averaging procedure we utilised can be found in the supplementary material, with more details about processing *P*(*f*) and *F*(*n*) functions. Using this approach, we were able to determine a single ensemble-averaged* S*(*f*), *P*(*f*), and *F*(*n*) for each type of acceleration and activity signal based on the 42 subjects; we examined the characteristics and scaling properties of these functions.

To examine the fine changes in the shape of the ensemble-averaged *S*(*f*) and *F*(*n*), we have taken their simple numerical derivate after log-transforming them, i.e., we determined the bin-by-bin slope of these functions on log scales. Considering *S*(*f*), the calculated slope is equal to –*β*, and is equal to *α* for *F*(*n*). We have converted these bin-by-bin slopes to *β* exponent values and plotted them as a function of frequency (for PSD) or box width (for fluctuation function). By examining these *β* exponent curves, we were able to assess to what extent and over which frequencies or box widths *S*(*f*) or *F*(*n*) exhibit 1/*f* noise (i.e., *β* ≈ 1). The technical description of calculating the *β* exponent curves can be found in the supplementary material.

To further describe the *S*(*f*) ∝ *1*/*f*^*β*^ and *F*(*n*) ∝ *n*^*α*^ scaling properties, we assessed the *β* and *α* exponents using two different approaches through linear fitting on the log-transformed *S*(*f*) and *F*(*n*), respectively. On the one hand, we performed the linear fitting on the ensemble-averaged *S*(*f*) and *F*(*n*) of each signal type, where we also indicated the goodness of the fit (*R*^2^) in addition to the value of *β* and *α* exponents. On the other hand, we performed linear fitting prior to ensemble-averaging on the noise-reduced PSDs and fluctuation functions (before the normalization step) from subject to subject from which we determined the mean of *β* and *α* exponents and their standard error (i.e., the standard deviation of the exponents divided by 42, the number of subjects) for every signal type.

### Ethics statement

The study was carried out as a part of research entitled “Examination neurobiological, cognitive and neurophenomenological aspects of the susceptibilities to mood swings or unusual experiences of healthy volunteer students„, and was approved by the Human Investigation Review Board, University of Szeged, Albert Szent-Györgyi Clinical Centre, Hungary (No 267/2018-SZTE) following its recommendations. All subjects gave written informed consent and the study was followed under the Declaration of Helsinki. All subjects were informed of their right to withdraw at any time without explanation and they were financially compensated.

## Results

In the following two sections, we present the results based on the analysis of the spectral densities and fluctuation functions ensemble-averaged over the 42 subjects first for the differently calculated activity signals and then for the variously preprocessed acceleration signals. In the presentation of our results, we show the corresponding figures for a few activity and acceleration signal types, the rest is available in the supplementary material. To make the resulting characteristics visually comparable, the *S*(*f*) and *F*(*n*) of the activity signals are visualized on the same scales as for the acceleration signals, even if the PSDs and fluctuation functions of the activity signals are evaluated in a narrower scale due to their significantly lower sampling rate.

### Spectral characteristics of the activity signals

#### Examining the PSDs of activity signals

**Figure 4:**
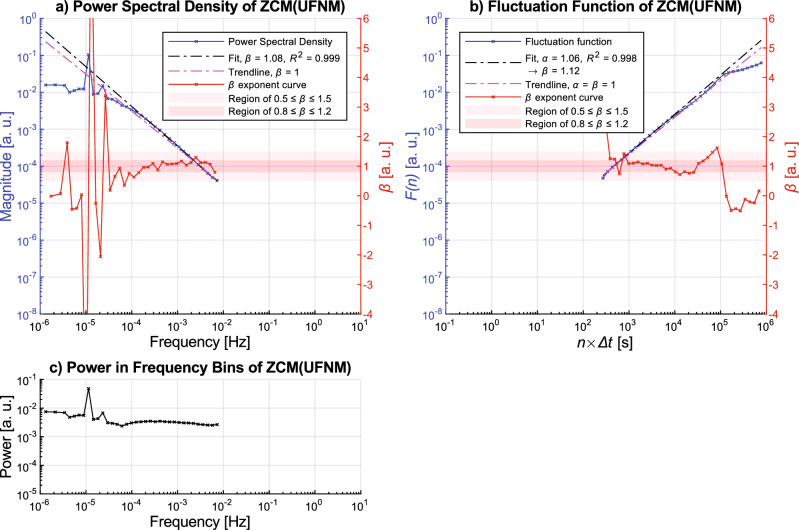
Ensemble-averaging-based results for the ZCM(UFNM) activity signals. In subplot (**a**), the curves associated with the left vertical axis (blue) are the following: ensemble-averaged power spectral density *S*(*f*) (crossed blue line), linear fit between 10^–4^ and 10^–2^ Hz on log(*S*(*f*)) versus log(*f*) (dash-dotted magenta line), 1/*f* trendline aligned to the 10^–2.5^ Hz component (dash-dotted black line). The *β* exponent curve appears as a crossed red line and it belongs to the right vertical axis (red) similar to the light red and deep red horizontal bands representing the loose and strict ranges mapped to the *β* exponent of the 1/*f* noise, respectively. On subplot b), the same marking is used as for subplot a), but in the case of the analysis of the ensemble-averaged fluctuation function *F*(*n*), where the linear fitting on log(*F*(*n*)) versus log(*n*) was executed between 10^2^ to 10^4^ s. In subplot c), the ensemble-averaged total power in log-spaced frequency bins *P*(*f*) is represented. The linear fitting based *β* and *α* exponents of the *S*(*f*) ∝ 1/*f*^*β*^ and *F*(*n*) ∝ *n*^*α*^ power-law scaling, while *R*^2^ value indicates the goodness of the fitting. Arrow symbol marks that the *β* exponent was calculated from *α* as *β* = 2 *α* − 1. Although the linear fittings were based on the intervals previously described, the line was drawn over the entire domain in both subplots (**a**) and (**b**).

The ZCM is one of the most common activity metrics, and in addition to PIM, the PSD of ZCM activity signals has already been investigated over a broader frequency range by a previous study^6^ revealing that they have 1/*f* characteristic over frequencies above the circadian rhythmicity. The study that discussed this also used epoch length of 60 s, however, other crucial aspects of the activity determination process are missing (e.g., the way of acceleration signal preprocessing, *T*_ZCM_ threshold level). In the light of these uncertainties, the question arises whether the 1/*f* characteristic is present in all the different types of ZCM activity signals or is only limited to specific acceleration preprocessings. Firstly, we are presenting our results in the case when the ZCM metric is applied to the unfiltered normalized magnitude of acceleration (i.e., ZCM(UFNM)) in Fig. 4.

Figure 4 shows the ensemble-averaged power spectral density *S*(*f*), fluctuation function *F*(*n*), and total power in log-spaced frequency bins *P*(*f*) derived from the ZCM(UFNM) activity signal of 42 subjects. As *S*(*f*) ∝ 1/*f*^*β*^ and *F*(*n*) ∝ *n*^*α*^ power-law relations appear linear on logarithmic scales, we discuss them exploiting this, so all functions are plotted on logarithmic scales. Considering *S*(*f*) in subplot a), a spectral characteristic emerges from the following components. A distinct change in the slope of the spectra can be observed at the corner frequency *f*_c_ of approximately 10^–4^ Hz. At frequencies higher than *f*_c_, the spectrum decays following the 1/*f* trendline. The scaling exponent *β* is 1.08 based on the slope of the linear fit between 10^–4^ Hz to 10^–2^ Hz on log(*S*(*f*)) versus log(*f*), which indicates the existence of scale-free nature and 1/*f* noise. Through the *β* exponent curve (introduced in the section “Materials and methods”), we visualized to what extent and in which frequency ranges the slope of the spectrum matches with the loose and strict ranges mapped to the *β* exponent of the 1/*f* noise. It can be seen that the *β* exponent curve is also within the tolerance range for 1/*f* noise, and *P*(*f*) is nearly constant in this 2-decade-long region as depicted in subplot c). At frequencies lower than *f*_c_, *S*(*f*) exhibits white noise as it breaks away from the 1/*f* trendline, and peaks are well-exposed at around the 24 and 12-h periodicities (around 10^–5^ Hz and 10^–4.8^ Hz, respectively). The fluctuation function presented in subplot b) also follows a trendline with a slope of 1, which we expected, given that the relationship between the scaling exponent of *S*(*f*) ∝ 1/*f*^*β*^ and *F*(*n*) ∝ *n*^*α*^ is defined as *β* = 2 *α* − 1. The linear fitting on log(*F*(*n*)) versus log(*n*) between 10^2^ to 10^4^ s reveals that the scaling exponent *α* is 1.06, from which *β* is 1.12. Another parallelism between *S*(*f*) and *F*(*n*) is that the fluctuation function also deviates from the trendline higher than a certain box width (i.e., in the region of low-frequency patterns) and flattens afterward. By examining the fluctuation function and the corresponding *β* exponent curve, it can be seen that there is a hump in *F*(*n*) at the box width of about 24 h. Our PSD-based spectral analysis results are consistent with the previously mentioned study^6^ which analysed the spectra of ZCM activity signals.

**Figure 5:**
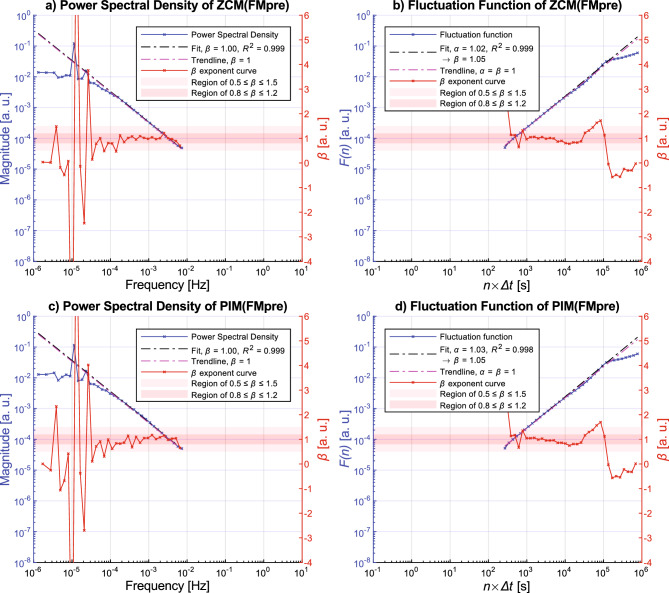
Ensemble-averaging-based results for the ZCM(FMpre) (subplots (**a**) and (**b**)) and PIM(FMpre) (subplots (**c**) and (**d**)) activity signals. For the description of the markings, see the caption of Fig. 4.

To examine the spectral properties of ZCM activity signals calculated from differently preprocessed acceleration data, Fig. 5 subplot (a) and (b) illustrate the result when the ZCM metric was combined with the FMpre (magnitude of the axially filtered acceleration) preprocessing technique instead of the UFNM. In contrast, Fig. 5. subplots (c) and (d) presents the result where the FMpre preprocessing was combined with a different activity metric (PIM). The resulting figures for the remaining 32 types of activity signals can be found in the supplementary material. By comparing Figs. 4 and Fig. 5, it is apparent that all three presented activity signal types generally follow the same spectral characteristics that we described above, independently of the chosen activity metric or acceleration signal preprocessing technique. The spectral shape of the PIM(FMpre) activity signals is also in alignment with what we found in the literature^56,57^ for the PIM activity metric. In the following, we will examine if activity signals produced by the other activity calculation methods have a similar spectral scale-free, 1/*f*-like characteristics in general as presented above.

#### Spectral comparison of different types of activity signals

**Figure 6:**
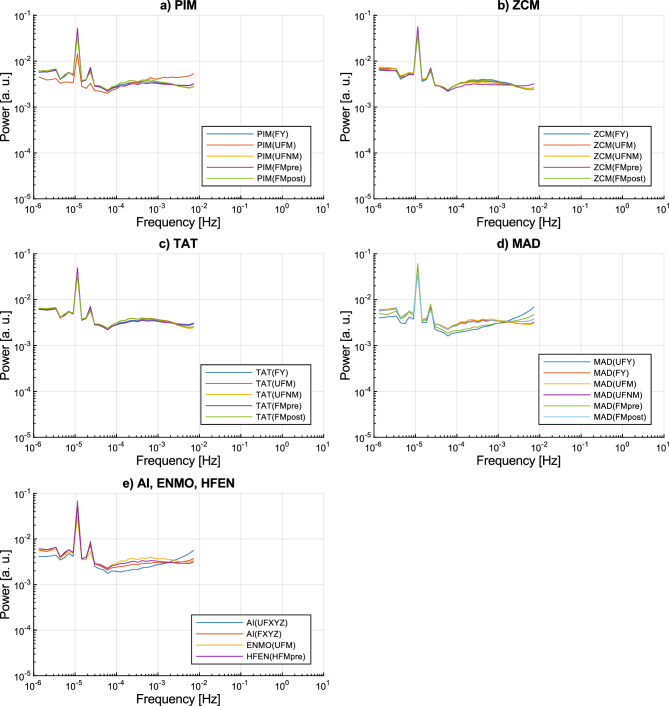
The ensemble-averaged total power in log-spaced frequency bins *P*(*f*) for each type of activity signal grouped by activity metrics. The last subplot (**e**) merges the AI, ENMO, and HFEN activity metric groups due to the few types of activity signals that can be produced with these metrics. To make it more clear, axial activity signals are represented only for the y axis (anteroposterior axis) as the *P*(*f*) curve of activity signals calculated from the different axes with the same activity determination method are generally following the same shape.

To illustrate how similar spectral characteristics the differently determined activity signals follow, Fig. 6 demonstrates the total power in log-spaced frequency bins *P*(*f*) for the different types of activity signals grouped into subplots by activity metrics. On one hand, the comparison of the *P*(*f*) curves within each subplot reveals the impact of the different acceleration preprocessing techniques on the spectral characteristic of the activity signals computed with the given metric. On the other hand, the discrepancies in the spectral characteristics caused by the different activity metrics can be assessed by comparing the subplots to each other. Note that although we have analysed 35 types of activity signals in total, Fig. 6 depicts only 25 types: if an activity metric could be applied to the axial acceleration signals separately, it is only represented in the case of the y axis (anteroposterior axis, i.e., FY or UFY acceleration signals) in the figure. The *P*(*f*) curve of the activity signal types determined along the x and z axes can be found in the supplementary material as they generally follow the same shape compared to those determined along the y axis.

As shown in Fig. 6, different types of activity signals can be described with the same spectral characteristic in general which we described above. In more detail, 29 of the 35 activity signal types have similar 1/*f* nature at frequencies higher than the corner frequency fc of approximately 10^–4^ Hz, only the last half decade shows slight variations. The remaining 6 activity signal types (PIM(UFM), AI(UFXYZ), MAD(FMpre), and MAD activity signals calculated from the unfiltered axial accelerations) have a slightly different spectral shape as their P(f) curves deviate from the horizontal line (i.e., they differ from the S(f) ∝  1/*f* scaling). However, all these differences can be explained. By definition, the MAD metric must be applied to the unfiltered magnitude of acceleration (UFM)^16^. If we calculate MAD activity signals according to the definition of this metric, the resulting signals follow the same spectral shape as the other 28 activity signal types. Even though there is no technical barrier of applying the MAD metric to axial acceleration signals or filtered acceleration signals, these combinations produce such activity signals whose spectral shape differs from the observed general characteristic. The inventors of the AI activity metric did not define whether their method requires filtered or unfiltered axial acceleration signals^17^. Nonetheless, if we apply the AI metric to filtered acceleration signals, it already results in activity signals whose spectral shapes are almost identical to the other 28 activity signal types. The PIM metric can be applied to UFM-type acceleration signals only if the resulting activity signal is corrected afterward^1^ (subtracting the integral of g), therefore, the way of correction could be the reason for the observed difference.

**Figure 7:**
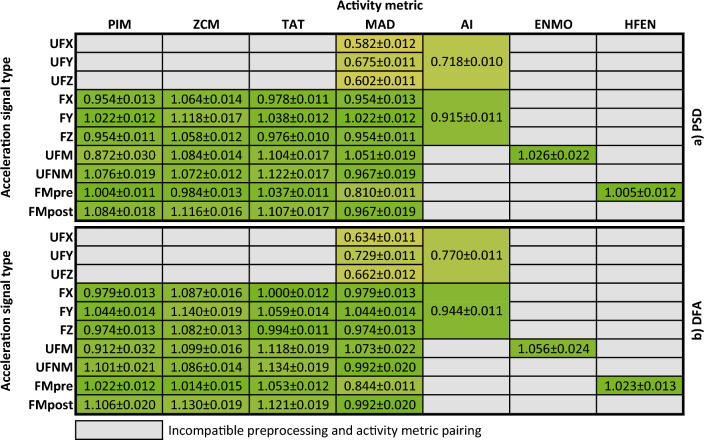
The mean of the *β* exponent values and the standard error for each type of activity signal based on the power spectral densities (**a**) and fluctuation functions (**b**) of the 42 subjects. The cells are interpreted as mean ± standard error, their colour is greener the closer the mean value is to 1. The colour of the cell linearly turns yellow as the mean values decreases to 0.3 or increases to 1.7.

So far, we have demonstrated the general 1/*f* nature by performing the linear fitting on the ensemble-averaged PSD (and fluctuation function) of the given activity signal type derived from the 42 subjects (e.g., Figs. 4 and 5). To statistically describe the strength of the 1/*f* nature, we also performed the linear fitting on the PSD (and fluctuation function) of a given type of activity signal for each of the 42 subjects and determined the mean and the standard error of the *β* exponent values (as detailed in the section “Materials and methods”). The technical parameters of the linear fitting were the same as before, the results can be seen in Fig. 7.

Even though all 35 activity signal types are shown in Fig. 7, the *β* exponents should be treated with caveats in the case of the PIM(UFM), AI(UFXYZ), MAD(FMpre), and MAD activity signals calculated from the unfiltered axial accelerations as the linear fitting was less accurate in their case due to the previously explained discrepancies. For the remaining 29 different types of activity signals, the values of the *β* exponents determined by both the PSD-based and fluctuation function-based linear fitting indicate clear 1/*f* fluctuations over the investigated frequency range and timescale as they fall between 0.8 and 1.2. Thus, from two independent perspectives, we showed that 1/*f* noise is a general feature of human motoric activity signals. Their spectral structure and notable frequency bands are also common. At frequencies lower than the corner frequency *f*_c_ of approximately 10^–4^ Hz, the spectrum exhibits white noise and the peaks associated with the 24 and 12-h periodicities arise, while at higher frequencies 1/*f* noise dominates.

### Spectral characteristics of the acceleration signals

#### Examining the PSDs of acceleration signals

As it was proved above, the spectral 1/*f* nature of human activity signals is independent of any given activity metric and is a general attribute. Consequently, the question arises whether acceleration signals can be described with the same spectral characteristics as activity signals, and if the acceleration signal preprocessing techniques have an impact on the observed characteristics. Note that the activity metrics are mostly nonlinear transformations, so it is not evident how similar are the PSDs of activity and acceleration signals. Moreover, 1/*f* noises exhibit unique properties and invariances in special cases for nonlinear operations^62^, which deserves further investigation on its own. Although a previous study^46^ has investigated the fluctuations of actigraphic acceleration signals, they only examined certain signal types in just a narrow frequency band and found 1/*f* fluctuations in that frequency range of signals recorded during wakefulness, but characteristics over a broader frequency range were not given. To investigate the general full-span spectral nature of acceleration signals, we analysed their PSDs in a frequency range of more than 6 decades using 10-day-long measurements. This spectral and DFA-based analysis were executed with the same methodology as for the activity signals. Note that it only makes sense to investigate the spectral characteristics for acceleration signal types where the last step of the preprocessing did not include digital filtering (i.e., UFX, UFY, UFZ, UFM, UFNM, FMpre, and HFMpre), otherwise we would observe the characteristics of the digital filter.

**Figure 8:**
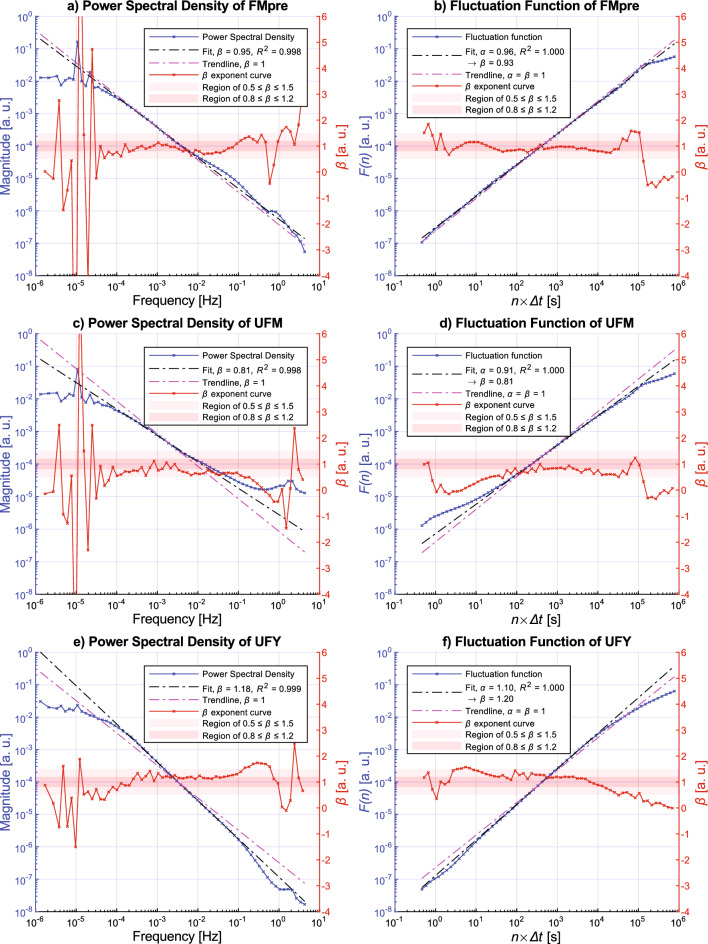
Ensemble-averaging-based results for the FMpre (subplots (**a**) and (**b**)), UFM (subplots (**c**) and (**d**)) and UFY (subplots (**e**) and (**f**)) acceleration signals. For the description of the markings used in this figure, see the caption of Fig. 4.

Since we began the presentation of our results with the activity signals, we will continue this top-down approach. Figure 8 presents and compares the spectral characteristics of 3 different types of acceleration signals that need to be preprocessed in substantially different ways. Subplot a) and b) characterizes the FMpre acceleration signal type, which requires the highest number of operations to generate (the triaxial acceleration is band-pass filtered before magnitude calculation). Note that Fig. 4 presents the characteristics of two activity signal types that use such acceleration signal preprocessing technique for the sake of comparability. The subplots c) and d) of Fig. 8 illustrate the spectral characteristics of the UFM acceleration signal type, which is the most straightforwardly preprocessed acceleration signal type (the magnitude of acceleration calculated from the raw triaxial acceleration data). Finally, out of the x, y, and z-axial projections of the acceleration vector recorded on the wrist of the subject, subplots e) and f) show the spectral characteristics of the UFY acceleration signal type (measured on the anteroposterior axis, parallel to the forearm).

If we narrow the frequency interval of the analysis to the same range where activity signals were interpreted (i.e., frequencies lower than approximately 10^–2^ Hz), Fig. 8 reveals that the spectral characteristics of these types of acceleration signal are very similar to what was previously shown for activity signals. Similarly, the slope of the PSDs changes at the corner frequency *f*_c_ of about 10^–4^ Hz. At frequencies lower than *f*_c_, the spectrums are flattened, and peaks are visible around the 24 and 12-h periodicities, although only the former peak is visible for the UFY signals. At frequencies higher than *f*_c_, the spectrums exhibit *S*(*f*) ∝ 1/*f*^*β*^ power-law scaling. However, the *β* exponent assessed by linear fitting between 10^–4^ Hz to 10^–2^ Hz slightly varies: while it is 0.95 for the FMpre, it is 0.81 in the case of the UFM and 1.18 for the UFY acceleration signals. As these *β* exponent values fall between 0.8 and 1.2, 1/*f* noise is observed for at least 2 decades for these acceleration signal types. A study has previously investigated UFM acceleration signals recorded during wakefulness and found that there is 1/*f* noise in the frequency range of 3.3 ⋅ 10^–4^ Hz to 3.3 ⋅ 10^–2^ Hz (approximately between 10^–3.5^ Hz and 10^–1.5^ Hz)^46^—which overlaps with the range where PSDs of our measurements exhibit the same nature. A slight difference is that the *β* exponent they found was closer to 1, but this could be explained by the fact that we did not only examine the wakeful segments of the recordings but analysed the 10-day-long acceleration signals as a whole. Examining the PSDs in Fig. 8 over that frequency range, which contains additional higher-frequency components compared to the activity signals because of the higher sampling rate (frequencies higher than approximately 10^–2^ Hz), there are notable differences. In the case of FMpre acceleration signals, the spectrum follows the 1/*f* trendline in that frequency range, too, despite the small local deviations that do not affect the global trend of the spectrum. In contrast, the *S*(*f*) of the UFM acceleration signals deviates from the *S*(*f*) ∝ 1/*f*^*β*^ power-law scaling by curving upwards at frequencies higher than 10^–2^ Hz, meanwhile the spectrum of the UFY acceleration signals curves downwards at frequencies higher than 10^–1^ Hz.

Although these observations are based on the PSDs, the fluctuation functions also reflect the described spectral characteristics. However, it is known that dominating periodicities create a frequency hump that masks the real shape of *F*(*n*) of long-correlated time series^46,63,64^ at around the box width equal to the period of the dominating periodicity. Moreover, a previous study^46^ has also noted that the estimation of power-law scaling is more reliable using PSDs, as linear fitting on the log-transformed fluctuation function could be easily biased by such humps. Compared to all the depicted characteristics (Figs. 4, Fig. 5, and Fig. 8), the ensemble-averaged *S*(*f*) of UFY acceleration signals has the smallest peak associated with 24-h periodicity. Therefore, its ensemble-averaged *F*(*n*) exhibits the least masking distortion, and therefore it flattens earliest, at the box width approximately equal to the reciprocal to the corner frequency *f*_c_ of *S*(*f*). This confirms our observation of the existence of a corner frequency based on spectral densities.

#### Spectral comparison of differently preprocessed acceleration signals

**Figure 9:**
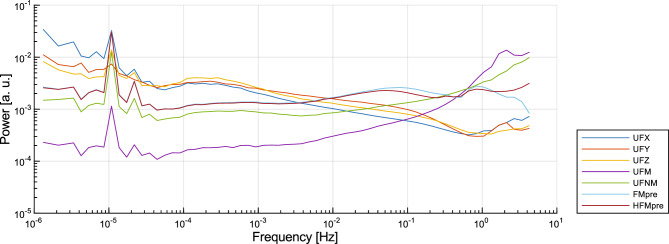
The ensemble-averaged total power in log-spaced frequency bins for each type of acceleration signal.

The HFMpre, UFNM, and the other two raw axial acceleration signals, UFX and UFZ, have not been discussed so far, their corresponding figures can be found in the supplementary material. Figure 9 presents the total power in each frequency bin *P*(*f*) for all the analysed acceleration signal types for the sake of comparability. The slope of the power curves clearly distinguishes the raw axial acceleration signals from the others.

Since the HFMpre acceleration signal can be considered as a variation of the FMpre acceleration signal (the only difference is that instead of a band-pass filter, a high-pass filter is used to condition the axial acceleration), they follow the same spectral characteristic, as expected. The UFNM acceleration signal is obtained by subtracting 1 g from the UFM data and taking the absolute value of the difference. The spectral shape of the UFNM acceleration signals follows the same characteristic as FMpre acceleration signals at frequencies lower than 10^–2^ Hz but differs from it at frequencies higher than 10^–2^ Hz. A relation can be established between the axial signal types depending on the strength of circadian rhythmicity observed in their *S*(*f*): while UFY has the smallest, UFX has the largest peak corresponding to 24-h periodicity. Moreover, UFX acceleration signals (measured on the mediolateral axis, perpendicular to the forearm) exhibit the most moderate flattening at frequencies lower than *f*_c_ among the raw axial acceleration signal types.

**Figure 10:**
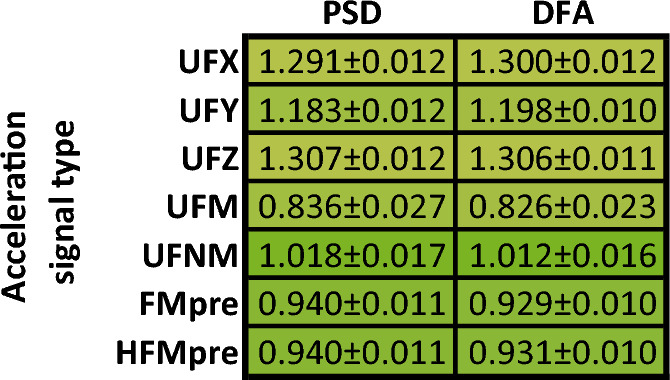
The mean of the *β* exponent values and the standard error for each type of activity signal based on the power spectral densities (left column) and fluctuation functions (right column) of the 42 subjects. The cells are interpreted as mean ± standard error, their colour is greener the closer the mean value is to 1. The colour of the cell linearly turns yellow as the mean values decreases to 0.3 or increases to 1.7.

To statistically describe the 1/*f* nature of the acceleration signals we executed the subject-based linear fitting to assess the mean of the *β* exponent values and its standard error for each acceleration signal type similar to what can be seen in Fig. 7 for the activity signals. The technical parameters of the linear fitting were the same as before, the results can be seen in Fig. 10.

For the raw axis acceleration signals (UFX, UFY, UFZ), the *β* exponent of the *S*(*f*) ∝ 1/*f*^*β*^ power-law scaling deviates most substantially from the ideal *β* = 1 to such an extent it is hard to state that these signals exhibit 1/*f* noise if speaking strictly. Note that, the range of 0.5 < *β* < 1.5 is also associated with 1/*f* noise in some fields^39^, in which case the axial acceleration signals also have 1/*f* nature. However, the raw axial acceleration signals are only projections of the acceleration vector, whose magnitude (UFM) has an undisputable 1/*f* nature as the *β* exponent meets the strict criterion for such noise (i.e., 0.8 < *β* < 1.2) in the examined 2-decade-long frequency range. The *β* exponent values of the other acceleration signal types generated by more complex preprocessing techniques (i.e., UFNM, FMpre, HFMpre) are even closer to the ideal *β* = 1. In summary, the magnitude of acceleration under all preprocessing conditions clearly exhibits 1/*f* noise even if we consider the strict criteria about the *β* exponent.

## Discussion

**Figure 11:**
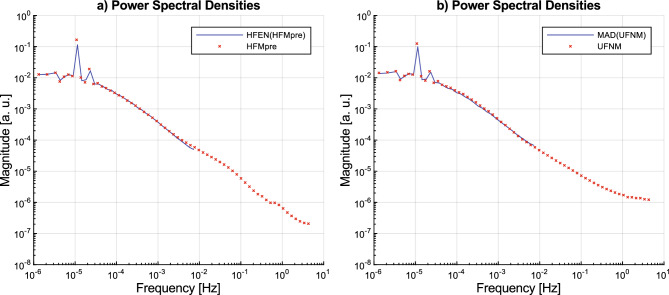
Ensemble-averaged PSD of acceleration signals versus activity signals calculated from them. Subplot (**a**) depicts the PSD of HFMpre acceleration signals versus the PSD of HFEN(HFMpre) activity signals. Subplot (**b**) depicts the PSD of UFNM acceleration signals versus the PSD of MAD(UFNM) activity signals.

As a result of our analysis, we found that both the differently computed activity signals and the acceleration signal preprocessed in various ways generally follow a universal spectral characteristic and have the same scale-free, 1/*f*-type nature, as Fig. 11 demonstrates, too. The spectral characteristics were investigated using independent time- and frequency-domain analysis methods (DFA, and PSD, respectively) prevalent in the literature, which yielded consistent results. We believe that this analytical approach of identifying the scale-free dynamics of daily human activity could be particularly advantageous, as it requires to take less methodological decisions compared to the statistical approach to the examination of statistical distributions of active/passive periods of activity signals^6–8,29–32^ that would otherwise may influence the results.

Based on our results and as Fig. 11 demonstrates, the activity calculation methods we analysed generally fulfil the requirement that they retain information related to long-term patterns despite the data compression, indicated by the substantial spectral agreement between acceleration and activity signals that was calculated from it on the low-frequency range of the spectra. It can be seen that the PSDs of acceleration signals follow the previously described characteristics even in the higher frequency range where the PSDs of activity signals are not containing information due to epoch-based data compression. However, in the last 1–2 decades of higher frequencies, there are unique differences between the spectra of the acceleration signals preprocessed in different ways, which might be explained by the fact that we are dealing with components related to fast-paced motions rather than daily activity patterns.

As an increasing number of actigraphic devices are able to store the raw acceleration signal, the question becomes relevant whether it is necessary to incorporate the calculation of activity values into the analysis of long-term motion patterns. The advantage of determining activity values is that the compressed actigraphic data (i.e., activity signals) can also store the necessary information as we have shown above, but it should be remembered that activity counting methods are not uniform, which creates further complications. Moreover, if our device is capable to measure and store the raw acceleration for a long enough time, we can obtain the necessary information from the raw data without losing the high-frequency information associated with rapid movements. Additionally, we retain the possibility to calculate activity at a later stage.

Related to the general spectral characteristics of human activity which we presented in the current work, the approximate positions of frequency bands with different spectral properties (i.e., the components of the observed characteristic) were estimated mainly on the basis of PSDs which were also reflected in the results of the DFA. However, further analysis could provide more information about the frequencies that delimit the assessed characteristics (e.g., the precise value of the corner frequency and its variance from subject to subject). Beyond technical-oriented investigations, it may also be beneficial to further examine and model biopsychological and neurophysiological mechanisms^48,56,65–67^ (i.e., notable periodicities beyond the circadian rhythm, such as the circatidal clock^68^ and the ultradian cyclicity^69^) to explore the underlying control mechanisms that are the causes of the observed characteristics of human activity.

Among the components of the general characteristics we have presented, the presence of 1/*f* noise over a certain frequency range was known for one type of acceleration and two types of activity signals. The presence of such noise in actigraphic measurements is often associated with the scale-independent^6,52,66^, fractal nature and complexity^33,35,51,56,57^ of the human activity, but detailed models about the appearance of 1/*f* noise have not been found. The relevance of such examinations is further enhanced by the fact that the presented scale-free nature and 1/*f*-type noise are not limited to human acceleration and activity signals. For example, scale-free fluctuations were also identified in human brain activity^65^, moreover the spectral characteristics of human activity presented in this work highly resemble what we have found for the PSD of minute-by-minute displacements in human location data^28^, including the transition between 1/*f* and white noise.

This transition in the characteristic we have shown has already been illustrated by other studies for two types of activity signals without further analysis or interpretation. The "whitening" of the 1/*f* noise—i.e., the disappearance of long-term correlations—can serve as a basis for future models. The deviation from the *S*(*f*) ∝ 1/*f*^*β*^ power-law scaling is observed even in the last 1–2 decades of higher frequencies for given types of acceleration signals, which has not been observed by others. Our comparison shows that these deviations are dependent on the preprocessing techniques, so it would be interesting to explore how these techniques distort the high-frequency information content of the acceleration signals. Based on these observations, the interpretation and modelling of the presented general spectral characteristics propose a number of open questions.

## Conclusion

To explore the general spectral nature of human activity in greater detail, we have performed PSD and DFA-based analysis on a dataset containing triaxial actigraphic acceleration signals of 42 healthy, free-living individuals. From the 10-day-long recording of a subject, we produced different types of acceleration and activity signals and characterized their ensemble-averaged PSDs and fluctuation functions over the entire frequency and box range.

Our main novel finding is that we revealed that the PSD of both the different types of actigraphic acceleration and activity signals generally follows a universal characteristic. At about 10^–4^ Hz (i.e., the corner frequency), a distinct change can be observed in slope of the spectrum on log–log scales. At frequencies higher than the corner frequency, the spectrum distinctly follows *S*(*f*) ∝ 1/*f*^*β*^ power-law scaling, where *β* ≈ 1. At frequencies lower than the corner frequency, the spectrum flattens and exhibits white noise, while two distinct peaks arise, belonging approximately to the 24- and 12-h periods (i.e., circadian and circatidal rhythmicity). The validity of our results was also supported by DFA, which yielded consistent outcomes.

Based on the general spectral characteristic of the different types of activity and acceleration signals we have analysed, we have proven that 1/*f* nature (i.e., the presence of 1/*f* noise for at least 2 decades) and the spectral scale-free property are not limited to a particular acceleration preprocessing technique or activity metric. Consequently, these spectral features are inherently existing in the multi-day motor activity of healthy, free-living humans and do not emerge at any particular stage of the activity determination process. Moreover, our results show that the measured raw acceleration signal has the same characteristics as the different activity signals calculated from it. The presence of 1/*f* noise in the acceleration signal was known for only a shorter period of wakefulness^46^, here we have generalized it by showing that the multi-day wrist movement of healthy humans exhibits 1/*f* noise above the frequency of the daily rhythmicity.

### Supplementary Information

Supplementary Information.

## Data Availability

The datasets used and/or analysed during the current study are available in the Figshare repository, 10.6084/m9.figshare.16437684.
