# Supplementary material for: General spectral characteristics of human activity and its inherent scale-free fluctuations
Source: Sci Rep. 2024 Jan 31;14:2604. doi: 10.1038/s41598-024-52905-8 (PMC10830482; doi:10.1038/s41598-024-52905-8)
Supplement: Supplementary file 1 — Supplementary Information. [file 41598_2024_52905_MOESM1_ESM.pdf]

# Supplementary information for manuscript entitled 'General spectral characteristics of human activity and its inherent scale-free fluctuations'

Bálint Maczák[1], Zoltán Gingl[1], and Gergely Vadai[1*]
[1] Department of Technical Informatics, University of Szeged, 6720, Szeged, Hungary

* Corresponding author
E-mail: vadaig@inf.u-szeged.hu

## Description of the calculation of Power Spectral Density (PSD)

Power Spectral Density (PSD) is used to determine the total power carried by the components of the analysed time series in a given frequency range $[f_1, f_2]$ while it also quantifies the correlation properties of the analysed time series based on its scaling properties. In order to calculate the spectral density, the analysed time series must first be transformed into the frequency domain, which can be done using the discrete Fourier transform (DFT) for sampled signals. The DFT is defined in Eq. (1), where $x_1, \dots x_N$ are the datapoints of the sampled signal $x$ with length $T$, while $k = 0, \dots, N-1$.

$$X[k] = \sum_{n=0}^{N-1} x[n] e^{-j\frac{2\pi kn}{N}} \quad (1)$$

The DFT calculation results in a complex vector $X$, that carries the amplitude and the phase information of each frequency component. The complex vector $X$ is two-sided as its 2nd half contains the complex conjugates of the elements in its 1st side except for $X[0]$ (i.e., DC-component). The single-sided PSD can be calculated based on the output of the DFT as seen below in Eq. (2), where $0 \leq k \leq N/2$.

$$S[k] = \begin{cases} |X[k]|^2 \cdot \frac{T}{N^2}, & k = 0 \\ 2|X[k]|^2 \cdot \frac{T}{N^2}, & k \neq 0 \end{cases} \quad (2)$$

The single-sided power spectral density's frequency scale is ranging from 0 Hz (DC-component) to $f_s/2$ with step size $\Delta f = 1/T$ (i.e., frequency resolution).

## Description of the Detrended Fluctuation Analysis (DFA) algorithm

Detrended Fluctuation Analysis (DFA) is a method that quantifies the correlation properties of the analysed time series based on the scaling properties of a so-called fluctuation function $F(n)$ to identify complex behaviour. For our analysis, we used a publicly available implementation [1] of DFA. The DFA algorithm determines the fluctuation function $F(n)$ according to the following algorithm. Let $X(t)$ be the time series to analyse that is consists $N$ datapoints.

1. Calculate the cumulative sum $Y(t)$ of the time series $X(t)$ as Eq. (3) defines below, where $\langle X \rangle$ is the mean of the time series $X(t)$.

$$Y(t) = \sum_{t'=1}^{t} (X(t') - \langle X \rangle) \tag{3}$$

2. Starting from the first datapoint of the cumulative sum $Y(t)$, split it into the maximum number of consecutive, non-overlapping boxes $s_1, \ldots, s_{N_n}$ that all consist of $n$ datapoints. If $N \,\%\, n \neq 0$, there will be a section at the end of the cumulative sum $Y(t)$ that does not belong to any box. To avoid this, the box-based splitting is repeated starting from the other end of the cumulative sum $Y(t)$ which yields a total of $2N_n$ boxes.
3. Using $p^{\text{th}}$ order polynomial fitting, calculate the $Y_s^{fit}$ local trend of the cumulative sum $Y(t)$ piecewise for each $s$ and then compute the variance as Eq. (4) defines below. For the polynomial fitting to be correct, $n \geq 4$ condition must be met. In the literature on human activity patterns, $p = 2$ is commonly used [2–4], this is called DFA-2 which we also used.

$$F^2(n,s) = \begin{cases} \frac{1}{n}\sum_{i=1}^{n}\left(Y\left((s-1)n+i\right) - Y_s^{fit}(i)\right)^2, & 1 \leq s \leq N_n \\ \frac{1}{n}\sum_{i=1}^{n}\left(Y(N-(s-N_n)n+i) - Y_s^{fit}(i)\right)^2, & N_n + 1 \leq s \leq 2N_n \end{cases} \tag{4}$$

4. Calculate the value of the fluctuation function $F(n)$ for $n$ width boxes as Eq. (5) defines below.

$$F(n) = \sqrt{\frac{1}{2N_n}\sum_{s=1}^{2N_n} F^2(n,s)} \tag{5}$$

5. The algorithm repeats these steps for different values $n$. In our analysis, the set of values $n$ was calculated to be logarithmically spaced between 4 and $N$ and to have approximately $B = 20$ values per decade. Accordingly, for the acceleration signals analysed, the minimum box width was 0.4 s due to the sampling rate of 10 Hz. Since the activity signals contained an activity value every 60 s, the minimum box width for activity signals was 4 minutes. As a consequence of logarithmic spacing, the fluctuation function $F(n)$ is equally spaced in $n$ if visualized on log-log axes.

# Calculation of ensemble-averaged spectral density and fluctuation functions

To determine the ensemble-averaged power spectral density $S(f)$ and fluctuation functions $F(n)$ of a specific acceleration or activity signal type, based on the 10-day-long actigraphic recording of the 42 subjects, we have implemented the following procedure.

The logarithmic-binning-based ensemble averaging procedure performs the same steps regardless of whether fluctuation functions or spectral densities are to be processed. It should be noted that the functions to be ensemble-averaged must be normalized in a preliminary step to ensure that their values cover a common scale. To achieve this, we performed sum-based normalization. Since calculating ensemble averages aim to determine the typical shape of the functions on a log-log scale, therefore, only function values for which the argument is greater than 0 are included in the normalization sum (i.e., we excluded the DC-component of the PSDs). The ensemble averaging procedure requires two parameters: the $N$ numbers of spectral densities or fluctuation functions, and $B$ which controls the resolution of the outputted ensemble average. For the sake of simplicity, let us consider spectral densities, the steps of the algorithm are the following.

1. For each spectral density $S(f)$, keep only those components for which $f > 0$ (i.e., omitting the DC component). In the case of fluctuation functions, this step has no effect since fluctuation function $F(n)$ is interpreted only for $n \geq 4$. The restriction of argument $n$ is previously justified in the DFA algorithm.
2. Calculate the broadest common frequency range $[f_{\min}, f_{\max}]$ of the spectral densities.
3. Split the frequency range $[f_{\min}, f_{\max}]$ into bins $b_1, \ldots, b_k$, such that their width is logarithmically spaced and each frequency decade is split across approximately $B$ bins. The $k$ value can be calculated based on Eq. (6).
$$k = [B(\log(f_{\max}) - \log(f_{\min}))] \quad (6)$$
4. Within each frequency bin, determine the average of the magnitude values of all spectral densities. This produces a single averaged magnitude value for each bin, which is assigned to the geometric mean of the given frequency bin. As a consequence, the resulting ensemble averaged spectral density consist of equally spaced components if visualized on log-log scale while the data reduction level is set by $B$.

If $N = 1$ (i.e., the input is a single $S(f)$ or $F(n)$), the above procedure performs averaging-based noise filtering instead of ensemble averaging. If analysing $1/f$ fluctuations through the scaling properties of spectral densities, it is also advantageous to calculate the sum in addition to the average in the 4$^{\text{th}}$ step. The summation results in the integral of each bin (i.e., the total power in the log-spaced frequency bins, $P(f)$), which must be constant in the case of $1/f$ noises. Since we used both

approaches in our analysis, let LBBA (Logarithmic-Binning Based Averaging) the procedure where averages are determined in the 4th step, and the procedure where sums are determined instead of averages will be referred to as LBBS (Logarithmic-Binning Based Summation) for the ease of differentiation.

Applying the procedures presented so far, the following processing chain is constructed to determine the ensemble-averaged spectral density $S(f)$, total power in log-spaced frequency bins $P(f)$, and fluctuation function $F(n)$ of a given type of acceleration or activity signal (the PIM(UFNM) activity signals were chosen as an example in Fig. S1) based on the 10-day-long actigraphic recording of 42 subjects.

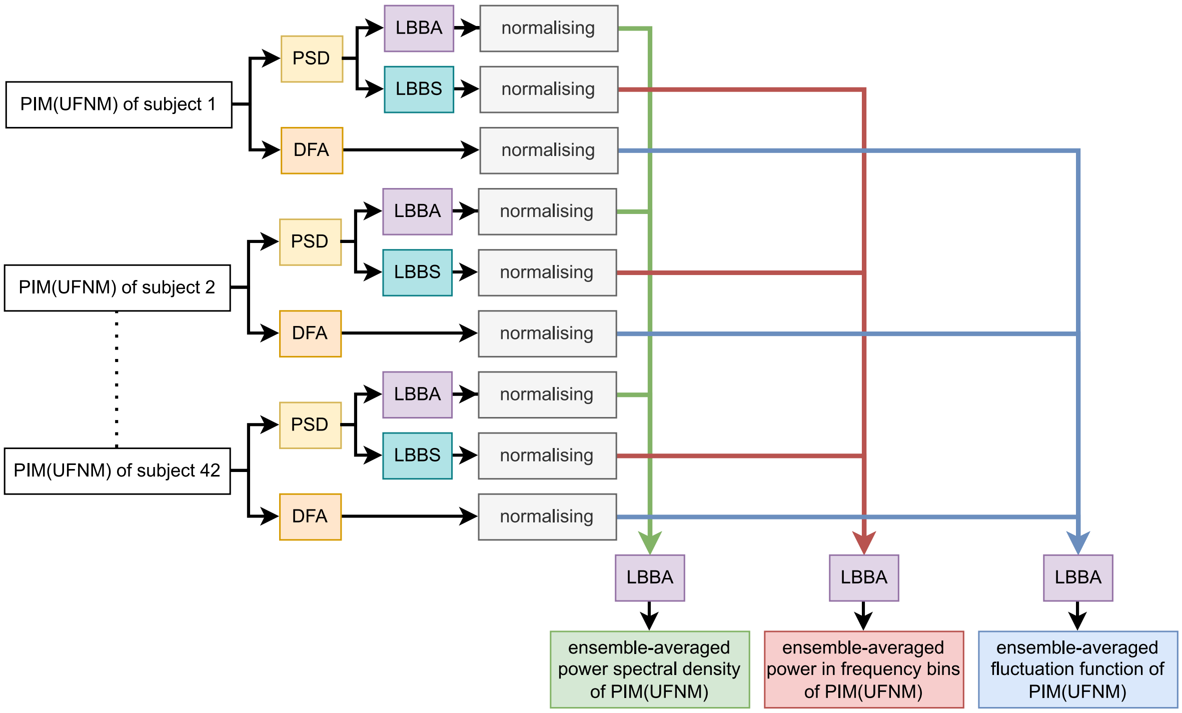


**Supplementary Figure S1. The processing chain of determining ensemble averages based on PSD and DFA analyses.**

In the processing chain, LBBA and LBBS operations immediately following the PSD calculation aim at noise reduction and to determine the total power in frequency bins beyond data reduction, respectively, both proceeded with $B$ = 100 resolution (i.e., 100 bins in each decade). After normalisation, the ensemble averaged spectral density and power in each frequency bin are determined using LBBA operation with $B$ = 10 resolution.

In the case of fluctuation functions, no intermediate step is required before normalisation, as fluctuation functions were already generated with $B$ = 20 resolution considering the computational resource requirements. The ensemble-averaged fluctuation function is determined afterward the

normalisation using LBBA operation with $B$ = 10 resolution to match the resolution of ensemble-averaged power spectral densities.

At the end of the process, for a given type of signal under investigation (PIM(UFNM) activity signal in Fig. S1 as an example) 3 ensemble-averages are obtained: power spectral density, total power in each log-spaced frequency bin, and fluctuation function; each with a resolution of 10 points per decade on logarithmic scale.

## Calculation of $\beta$ exponent curves

To describe the $S(f) \propto 1/f^{\beta}$ and $F(n) \propto n^{\alpha}$ scaling properties, the $\beta$ and $\alpha$ exponents can be evaluated between every consecutive point of the ensemble-averaged spectral density and fluctuation function based on the numerical derivation of the log-transformed data. The $\beta$ exponent between each successive point is calculated using Eq. (7), where $y_{\mathrm{PSD}}$ is the magnitude values of the $N$-long ensemble-averaged spectral density and $x_{\mathrm{PSD}}$ is the corresponding frequencies, while $i = 1, \ldots, N - 1$.

$$\beta[i] = -\frac{\log(y_{\mathrm{PSD}}[i+1]) - \log(y_{\mathrm{PSD}}[i])}{\log(x_{\mathrm{PSD}}[i+1]) - \log(x_{\mathrm{PSD}}[i])} \tag{7}$$

If we exploit that $\beta = 2\alpha - 1$, we can calculate the $\beta$ exponents based on the $\alpha$ exponents evaluated between every consecutive point of the ensemble-averaged fluctuation functions. Eq. (8) defines this, where $y_{\mathrm{DFA}}$ is the fluctuation values of the $N$-long ensemble-averaged fluctuation function and $x_{\mathrm{DFA}}$ is the corresponding box widths, while $i = 1, \ldots, N - 1$.

$$\beta[i] = 2\left(\frac{\log(y_{\mathrm{DFA}}[i+1]) - \log(y_{\mathrm{DFA}}[i])}{\log(x_{\mathrm{DFA}}[i+1]) - \log(x_{\mathrm{DFA}}[i])}\right) - 1 \tag{8}$$

Using the two formulas results in two exponent curves that describe the scaling properties of a given signal type in relatively high resolution compared to common linear fitting.

# Further resulting figures of the general spectral characterization of activity and acceleration signals

In this appendix, we are presenting the rest of the figures following the same top-down approach as in the manuscript, i.e., firstly we are depicting the resulting figures illustrating the general spectral characteristics in the case of the activity signals, then for acceleration signals.

## *Activity signals*

### ZCM

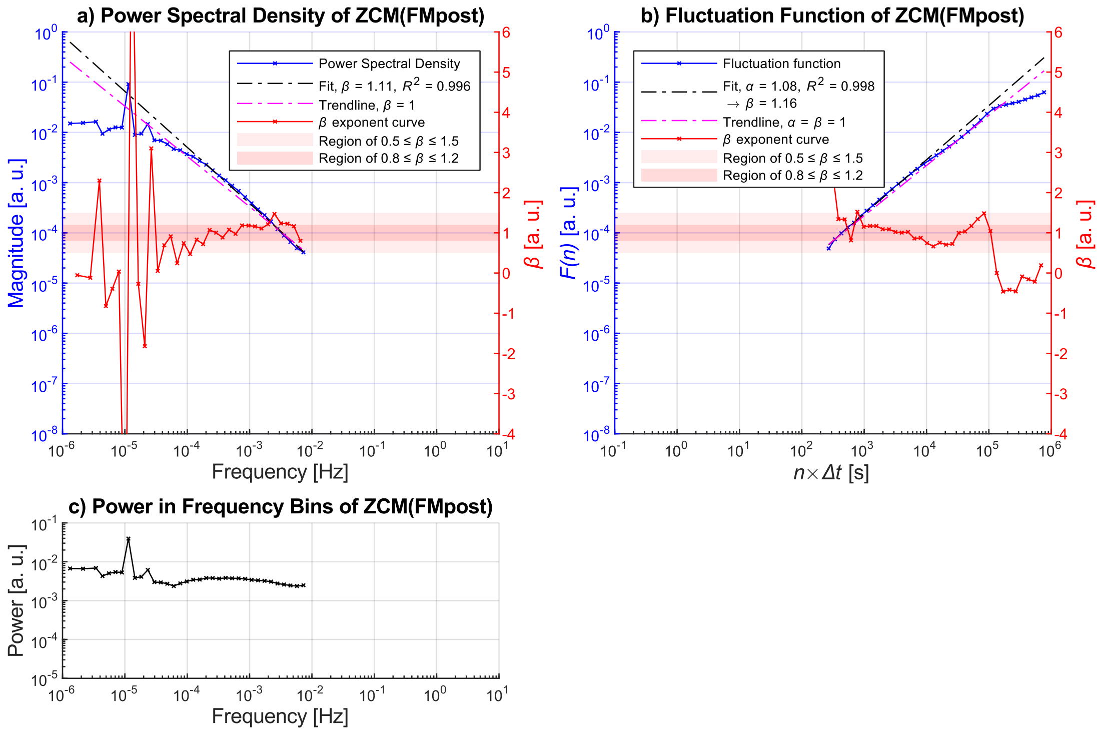


**Supplementary Figure S2. Ensemble-averaging-based results for the ZCM(FMpost) activity signals.** In subplot a), the curves associated with the left vertical axis (blue) are the following: ensemble-averaged power spectral density $S(f)$ (crossed blue line), linear fit between $10^{-4}$ Hz to $10^{-2}$ Hz on $\log(S(f))$ versus $\log(f)$ (dash-dotted magenta line), $1/f$ trendline aligned to the $10^{-2.5}$ Hz component (dash-dotted black line). The $\beta$ exponent curve appears as a crossed red line and it belongs to the right vertical axis (red) similar to the light red and deep red horizontal bands representing the loose and strict ranges mapped to the $\beta$ exponent of the $1/f$ noise, respectively. On subplot b), the same marking is used as for subplot a), but in the case of the analysis of the ensemble-averaged fluctuation function F(n), where the linear fitting on $\log(F(n))$ versus $\log(n)$ was executed between $10^2$ s to $10^4$ s. In subplot c), the ensemble-averaged total power in log-spaced frequency bins $P(f)$ is represented. The linear fitting based $\beta$ and $\alpha$ exponents of the $S(f) \propto 1/f^{\beta}$ and $F(n) \propto n^{\alpha}$ power-law scaling, while $R^2$ value indicates the goodness of the fitting. Arrow symbol marks that the $\beta$ exponent was calculated from $\alpha$ as $\beta = 2\,\alpha - 1$. Although the linear fittings were based on the intervals previously described, the line was drawn over the entire domain in both subplots a) and b).

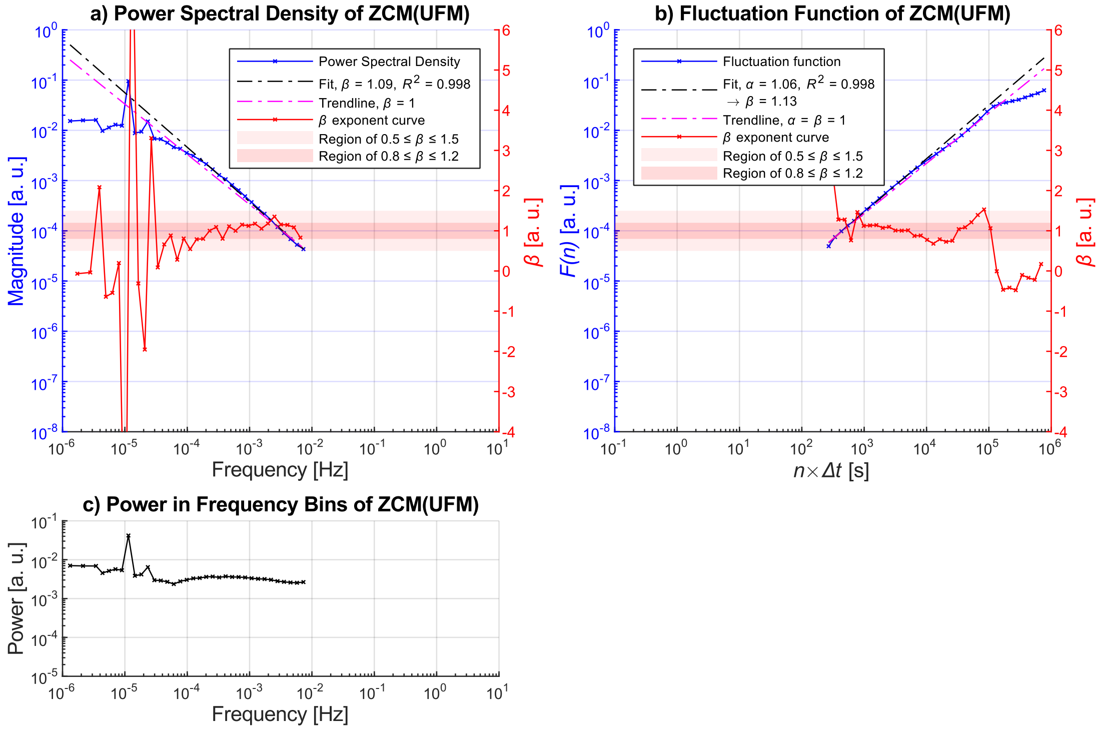


**Supplementary Figure S3. Ensemble-averaging-based results for the ZCM(UFM) activity signals.** For the description of the markings, see the caption of Supplementary Fig. S2.

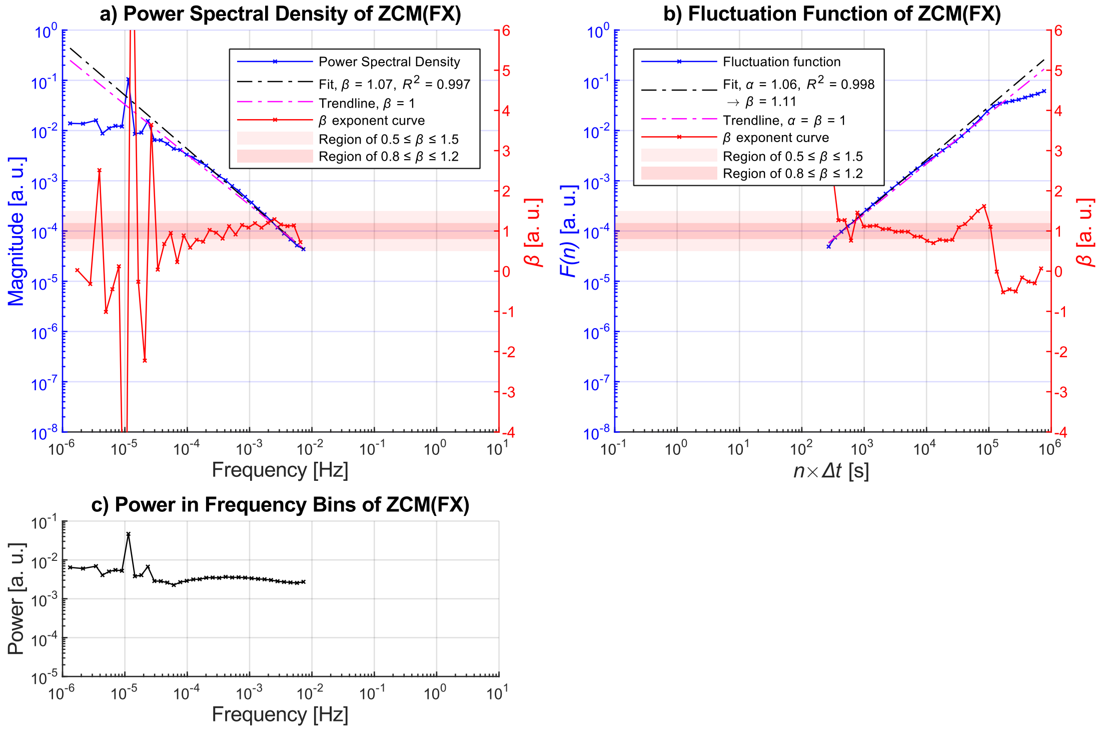


**Supplementary Figure S4. Ensemble-averaging-based results for the ZCM(FX) activity signals.** For the description of the markings, see the caption of Supplementary Fig. S2.

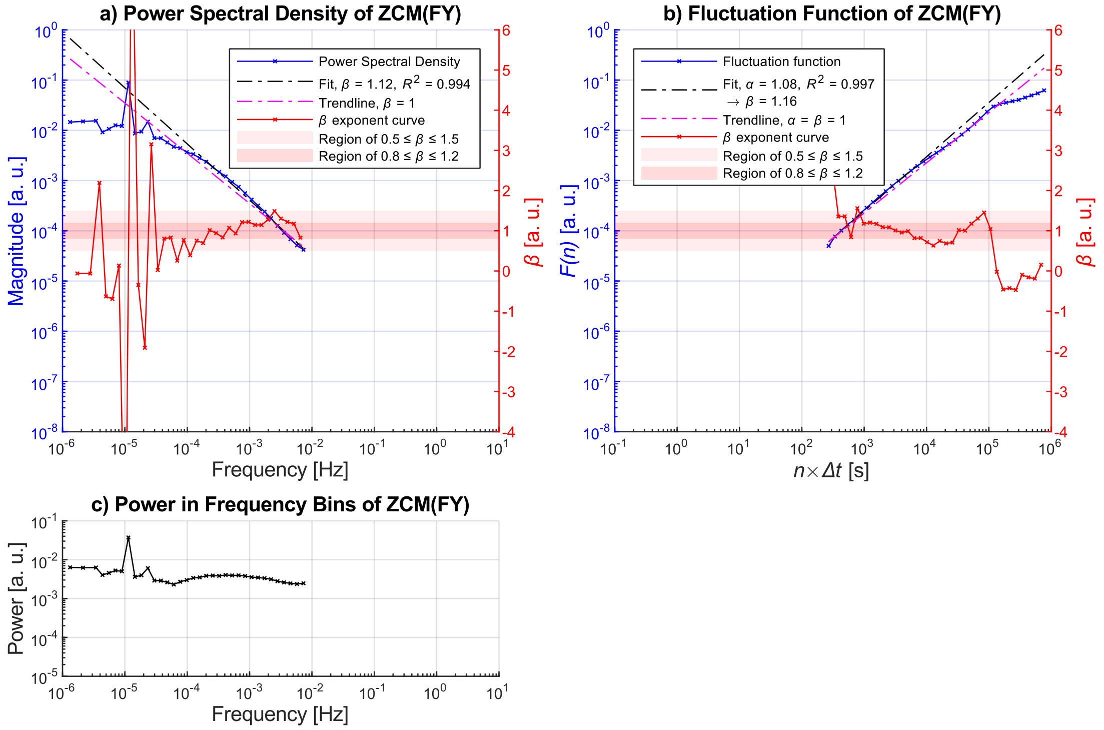


**Supplementary Figure S5. Ensemble-averaging-based results for the ZCM(FY) activity signals.** For the description of the markings, see the caption of Supplementary Fig. S2.

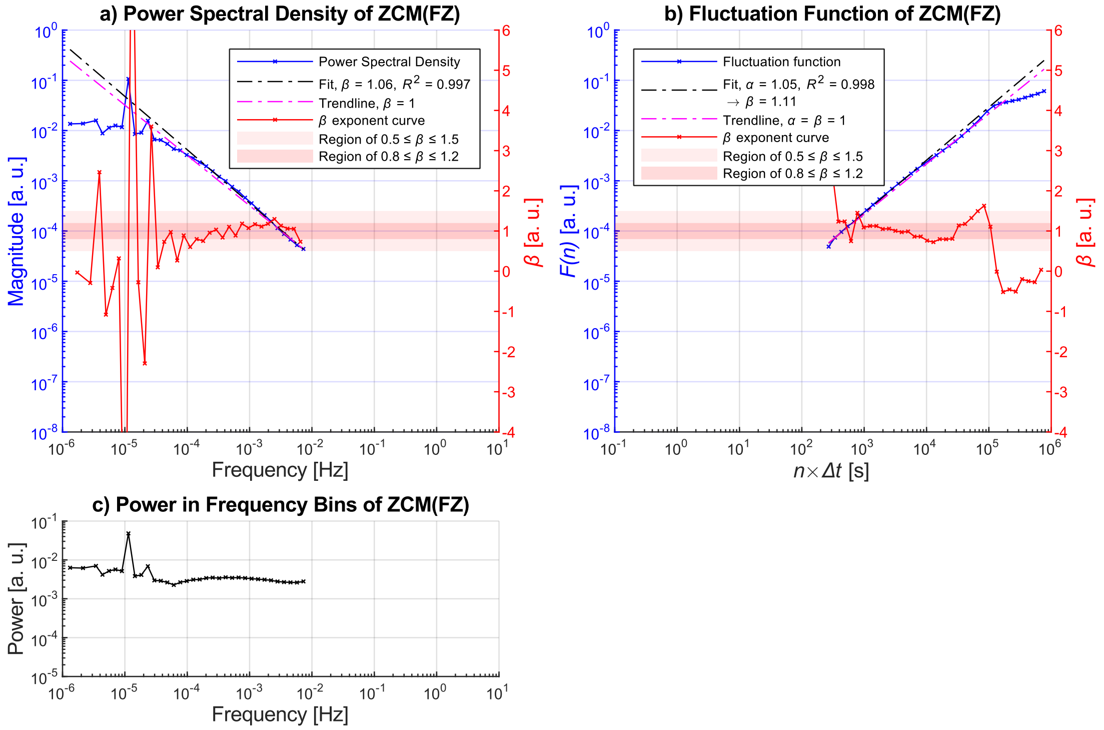


**Supplementary Figure S6. Ensemble-averaging-based results for the ZCM(FZ) activity signals.** For the description of the markings, see the caption of Supplementary Fig. S2.

## TAT

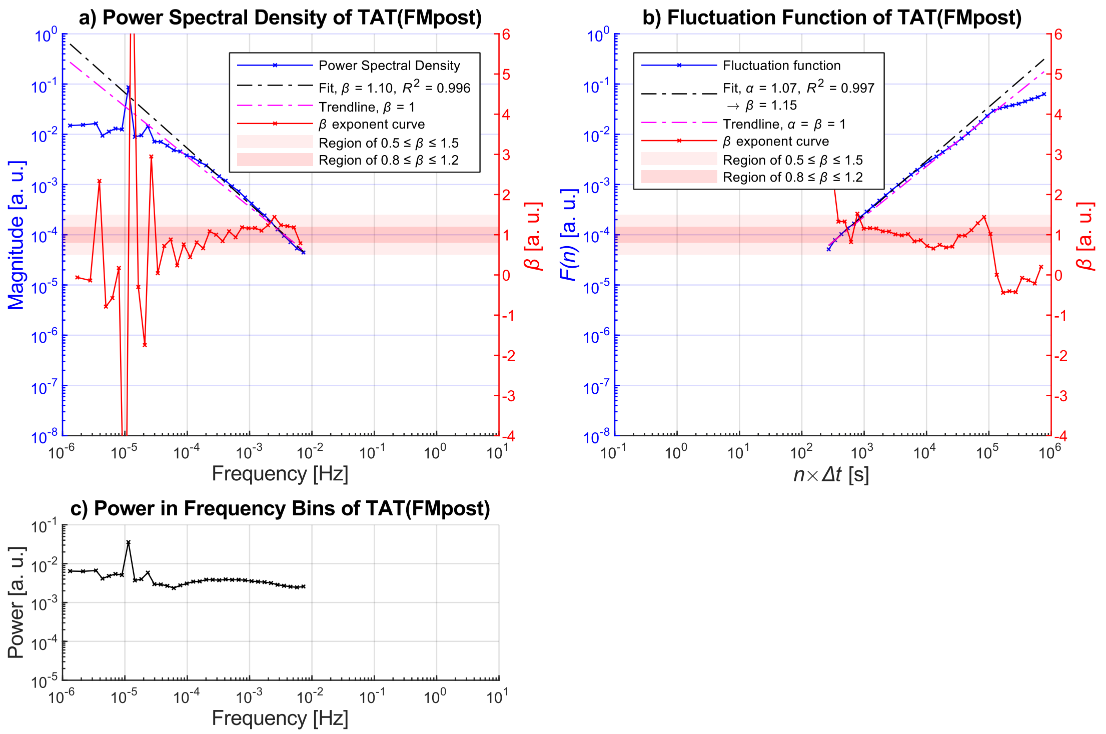


**Supplementary Figure S7. Ensemble-averaging-based results for the TAT(FMpost) activity signals.** For the description of the markings, see the caption of Supplementary Fig. S2.

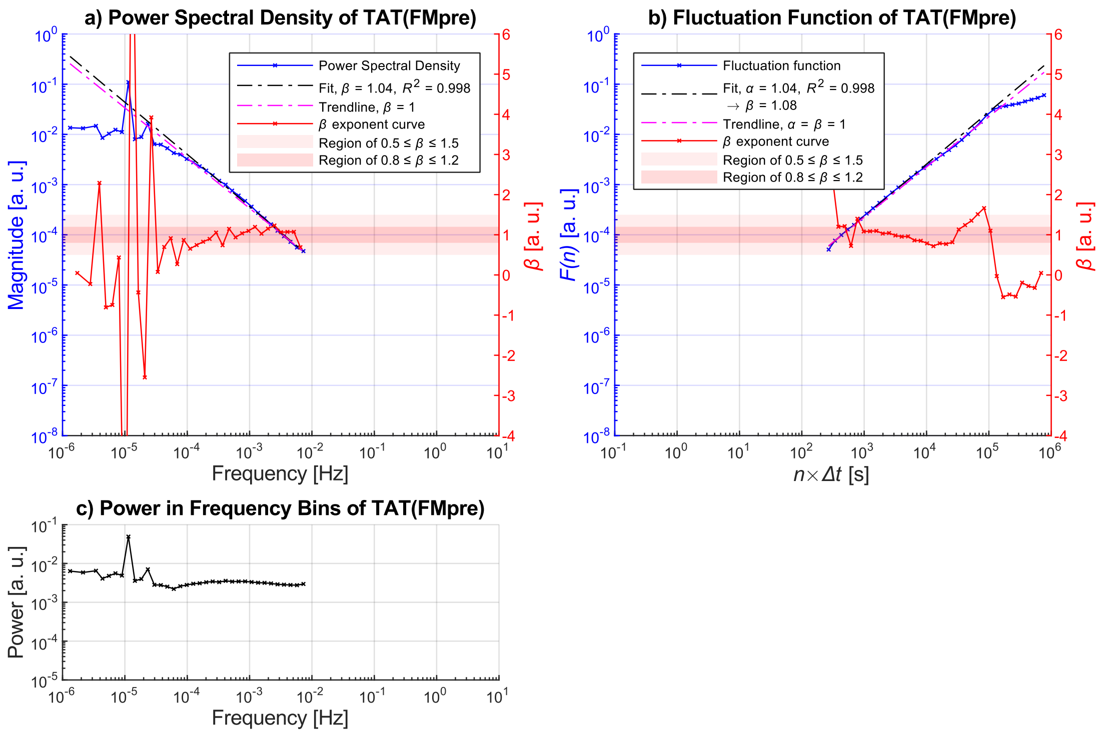


**Supplementary Figure S8. Ensemble-averaging-based results for the TAT(FMpre) activity signals.** For the description of the markings, see the caption of Supplementary Fig. S2.

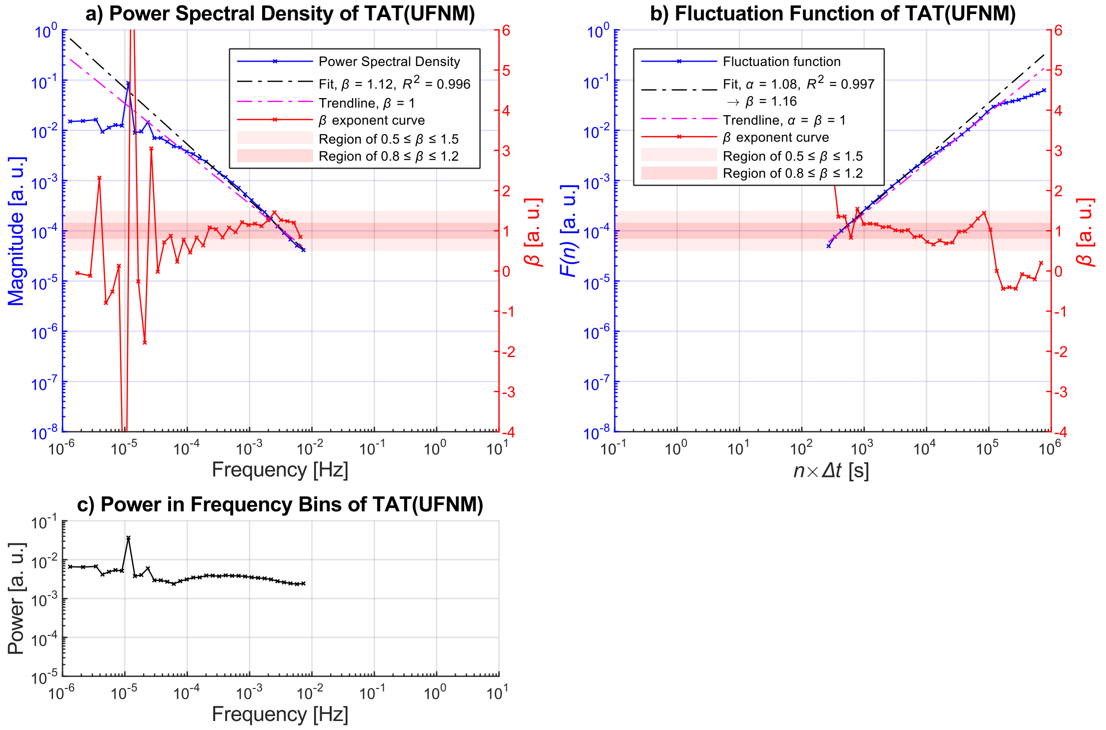


**Supplementary Figure S9. Ensemble-averaging-based results for the TAT(UFNM) activity signals.** For the description of the markings, see the caption of Supplementary Fig. S2.

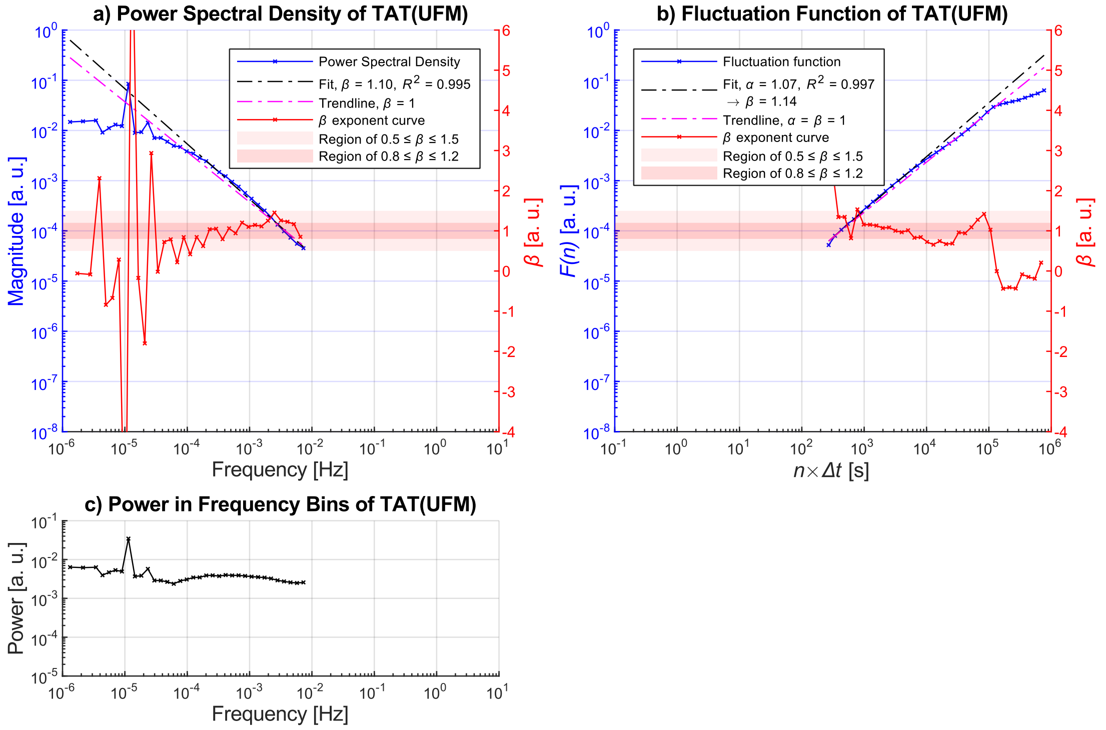


**Supplementary Figure S10. Ensemble-averaging-based results for the TAT(UFM) activity signals.** For the description of the markings, see the caption of Supplementary Fig. S2.

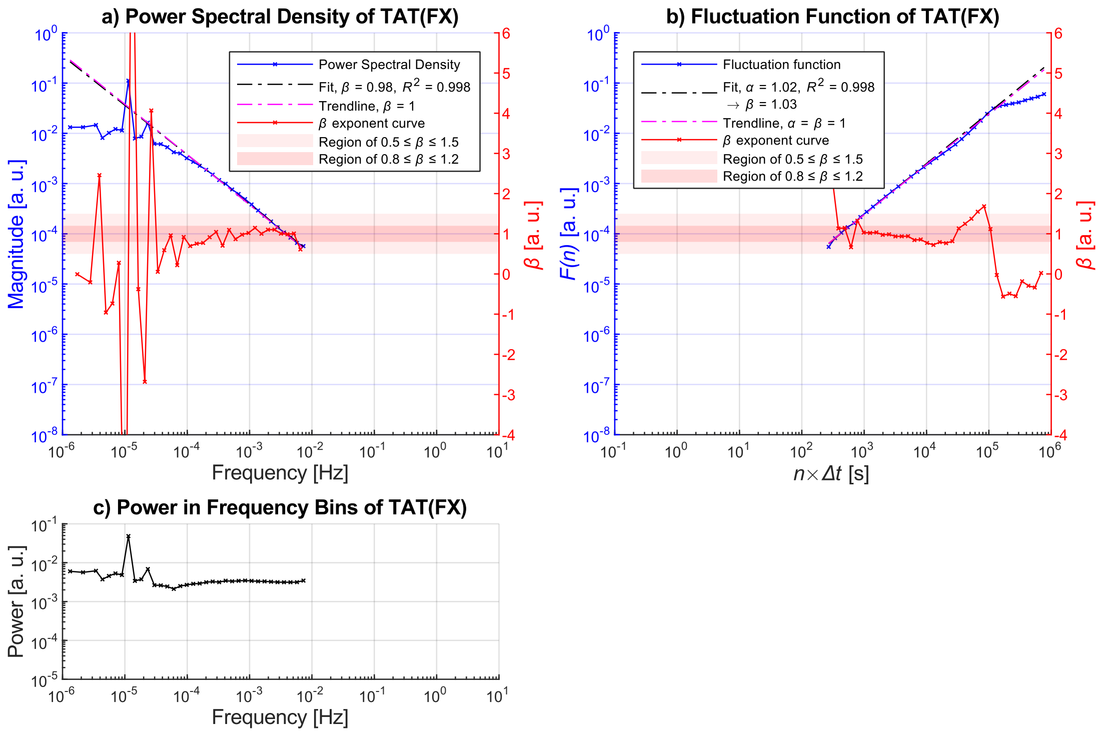


**Supplementary Figure S11. Ensemble-averaging-based results for the TAT(FX) activity signals.** For the description of the markings, see the caption of Supplementary Fig. S2.

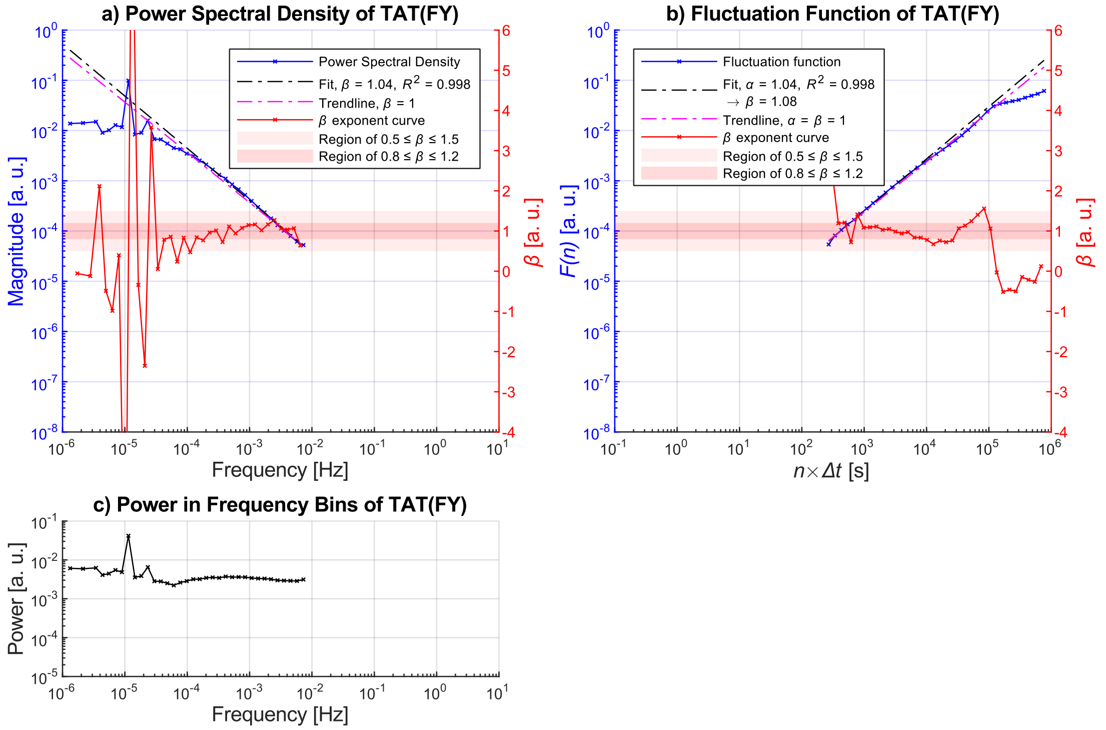


**Supplementary Figure S12. Ensemble-averaging-based results for the TAT(FY) activity signals.** For the description of the markings, see the caption of Supplementary Fig. S2.

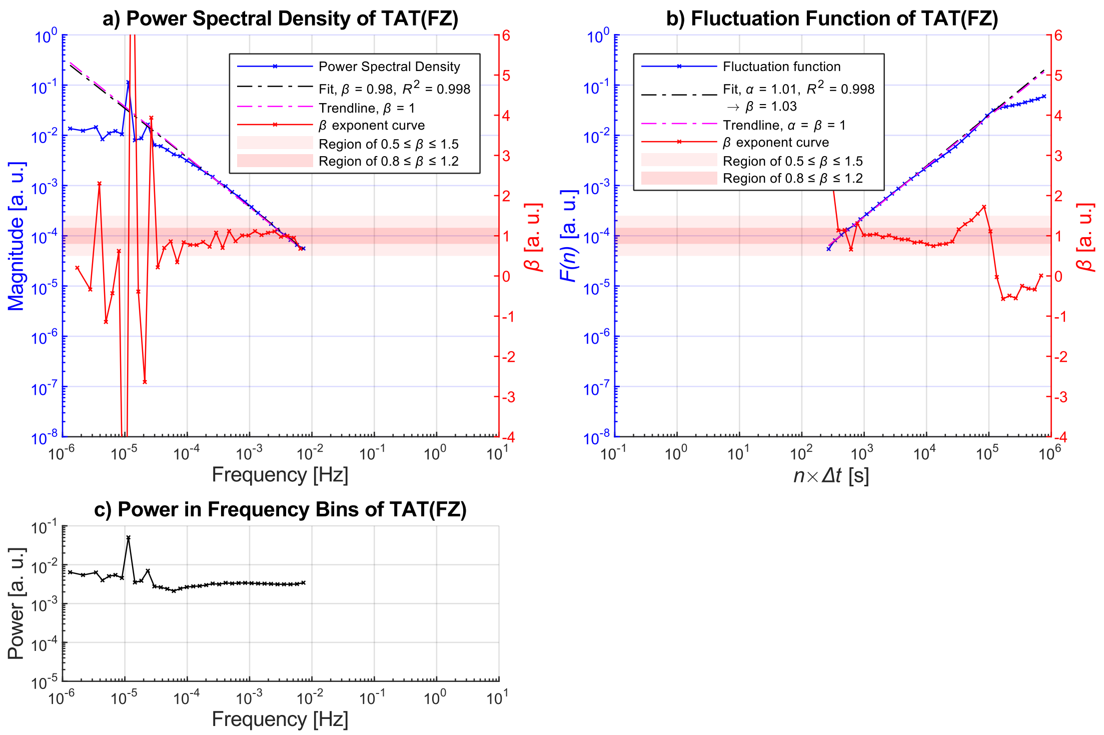


**Supplementary Figure S13. Ensemble-averaging-based results for the TAT(FZ) activity signals.** For the description of the markings, see the caption of Supplementary Fig. S2.

PIM

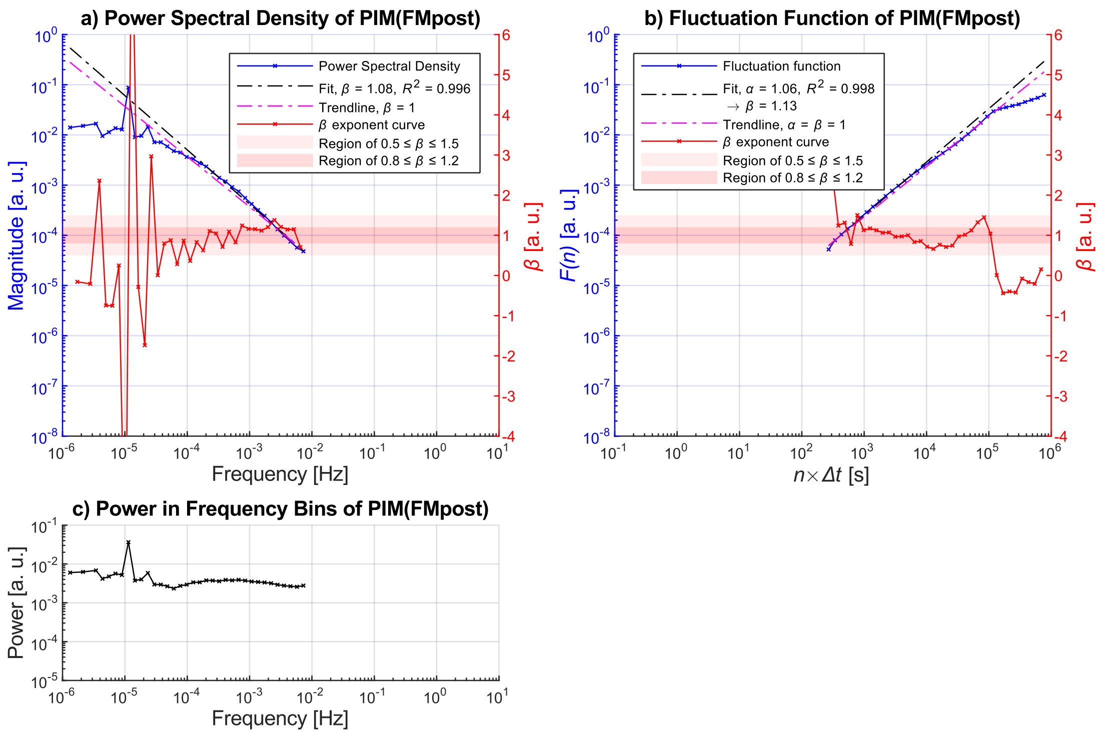


**Supplementary Figure S14. Ensemble-averaging-based results for the PIM(FMpost) activity signals.** For the description of the markings, see the caption of Supplementary Fig. S2.

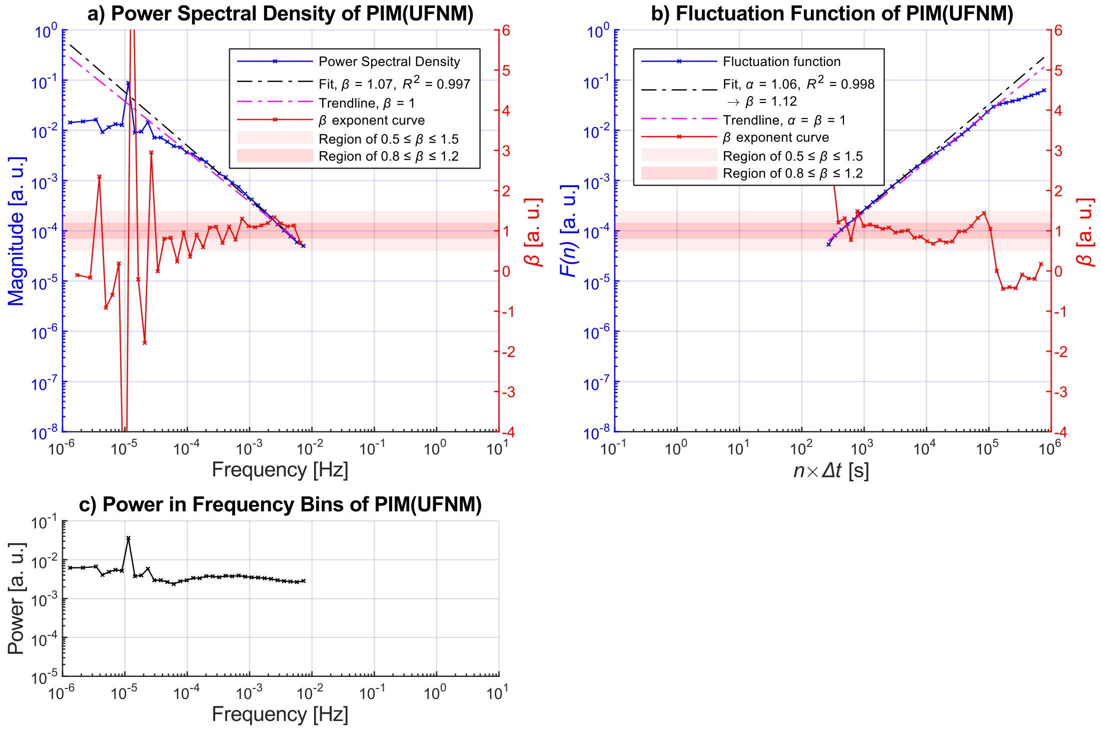


**Supplementary Figure S15. Ensemble-averaging-based results for the PIM(UFNM) activity signals.** For the description of the markings, see the caption of Supplementary Fig. S2.

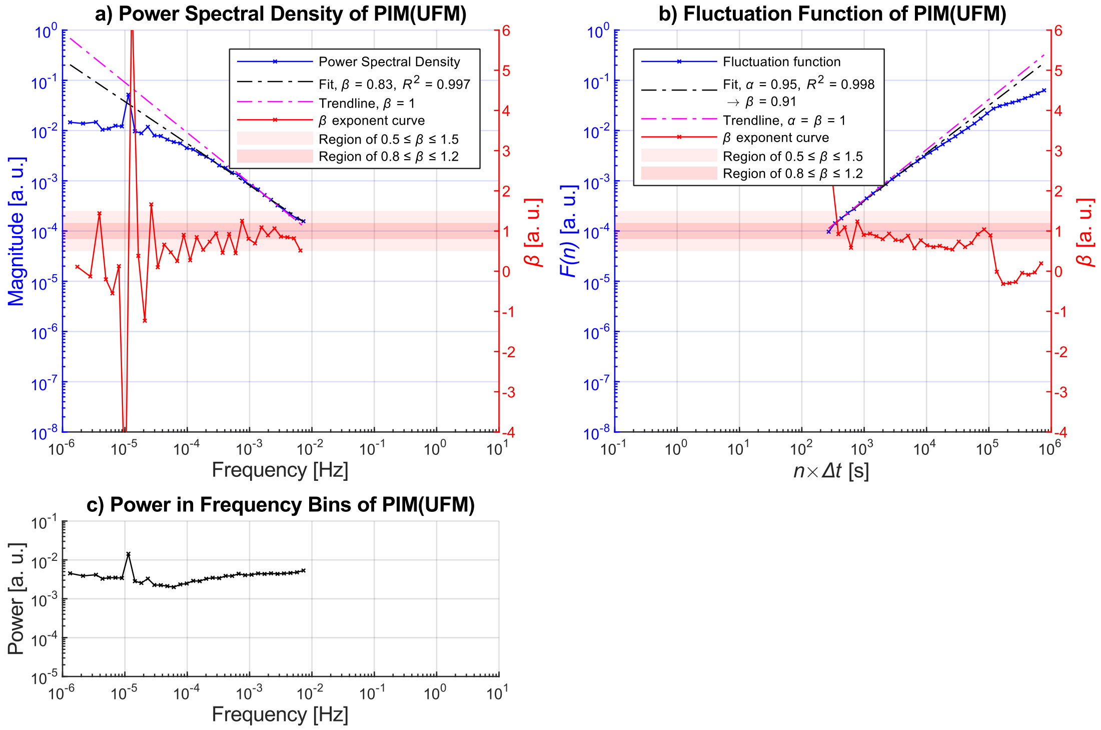


**Supplementary Figure S16. Ensemble-averaging-based results for the PIM(UFM) activity signals.** For the description of the markings, see the caption of Supplementary Fig. S2.

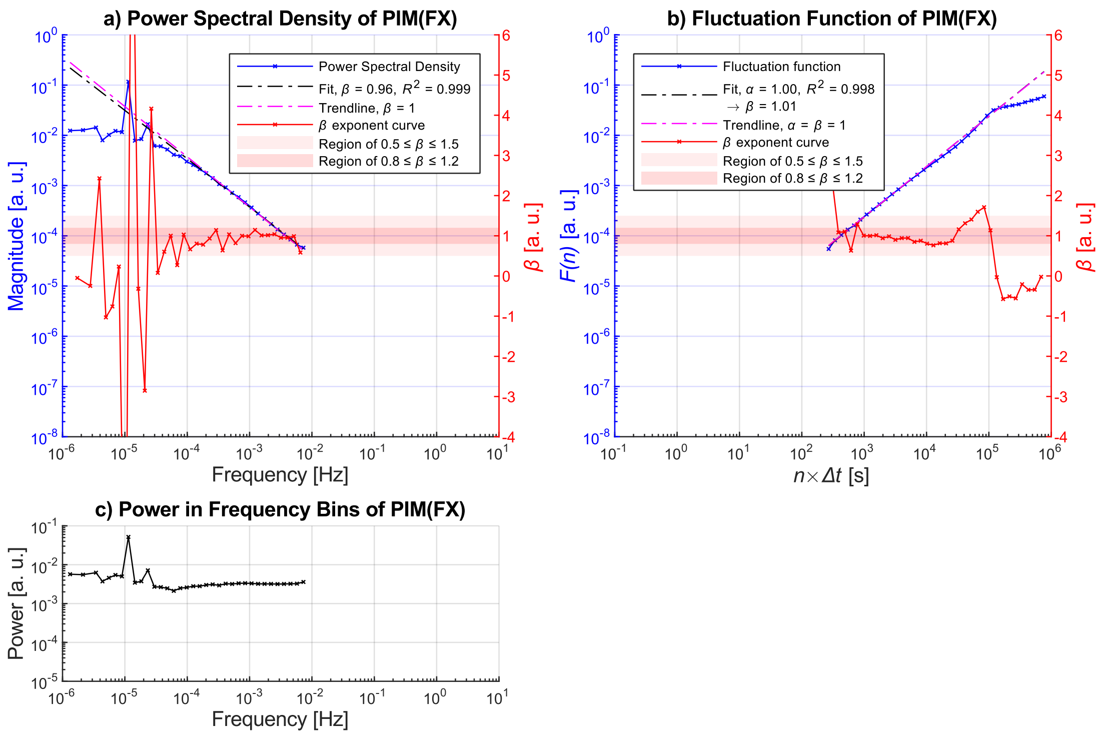


**Supplementary Figure S17. Ensemble-averaging-based results for the PIM(FX) activity signals.** For the description of the markings, see the caption of Supplementary Fig. S2.

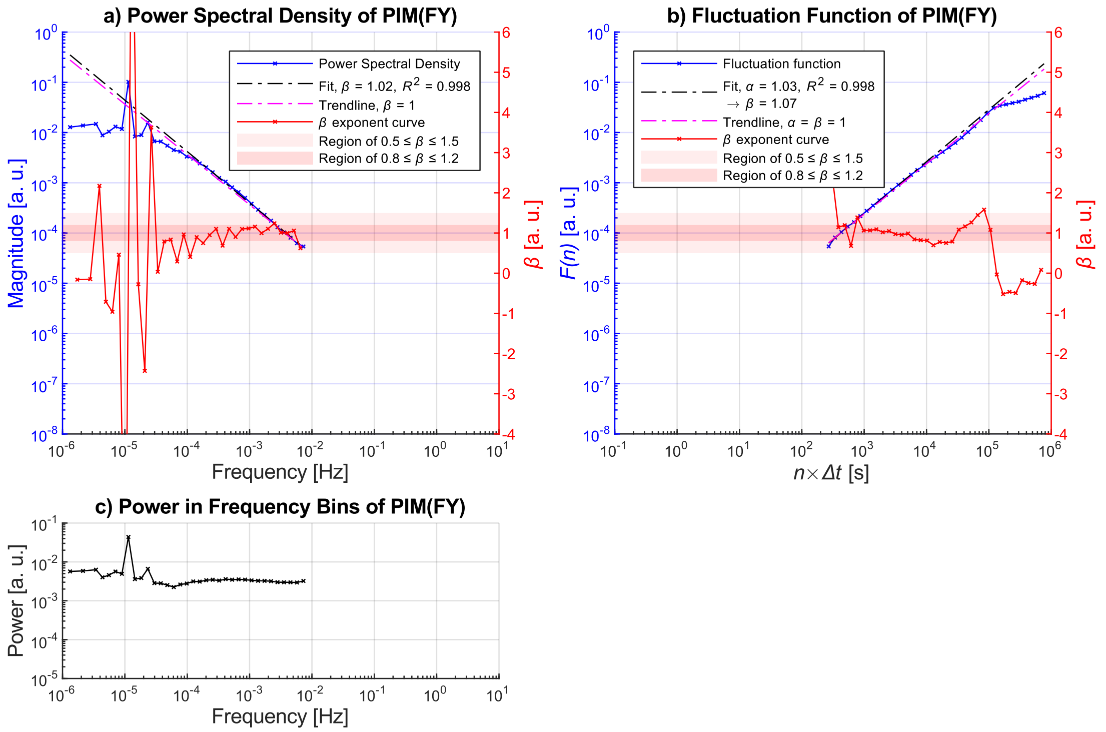


**Supplementary Figure S18. Ensemble-averaging-based results for the PIM(FY) activity signals.** For the description of the markings, see the caption of Supplementary Fig. S2.

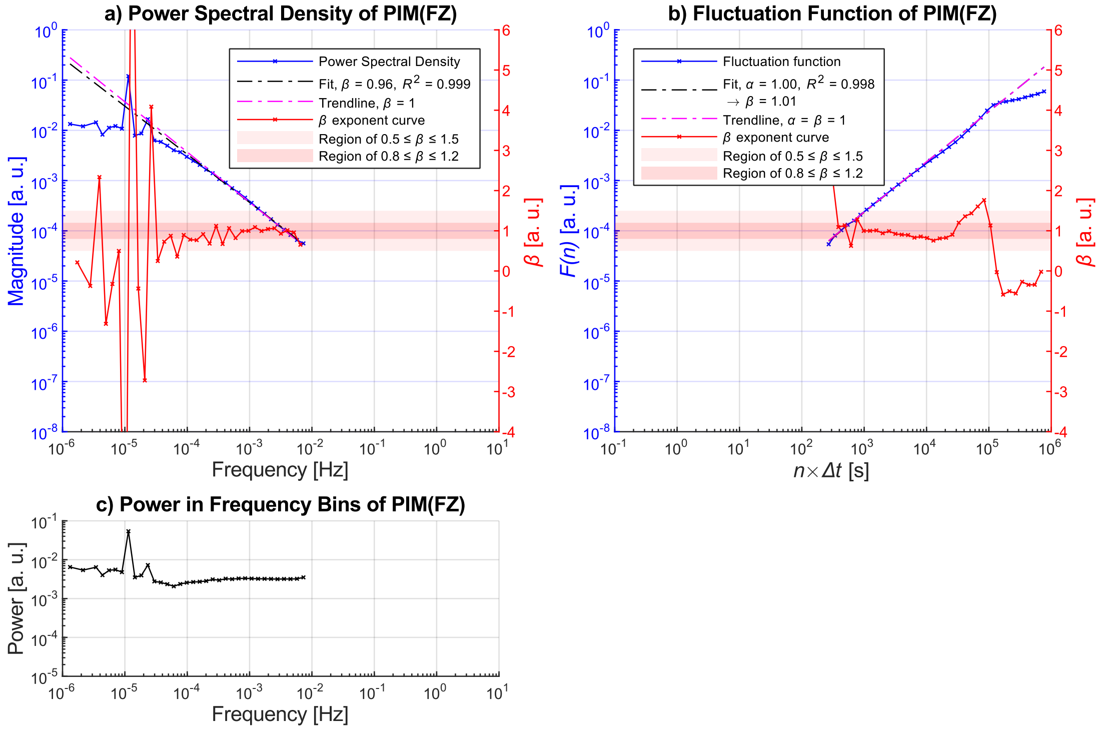


**Supplementary Figure S19. Ensemble-averaging-based results for the PIM(FZ) activity signals.** For the description of the markings, see the caption of Supplementary Fig. S2.

## MAD

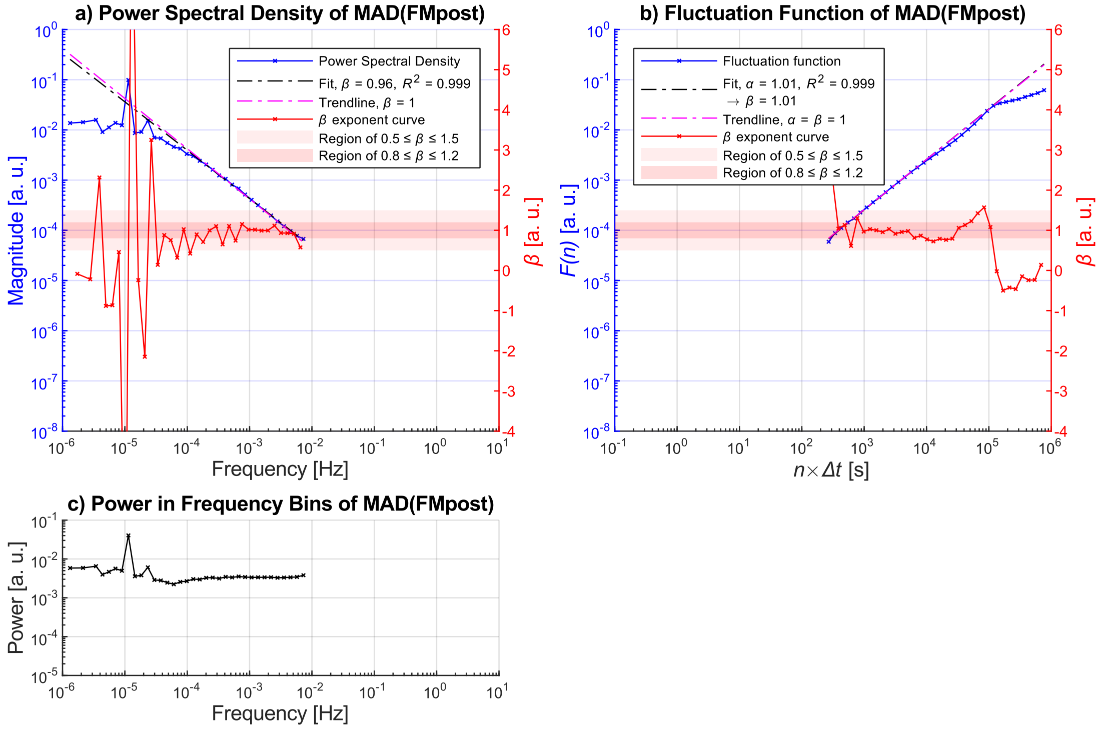


**Supplementary Figure S20. Ensemble-averaging-based results for the MAD(FMpost) activity signals.** For the description of the markings, see the caption of Supplementary Fig. S2.

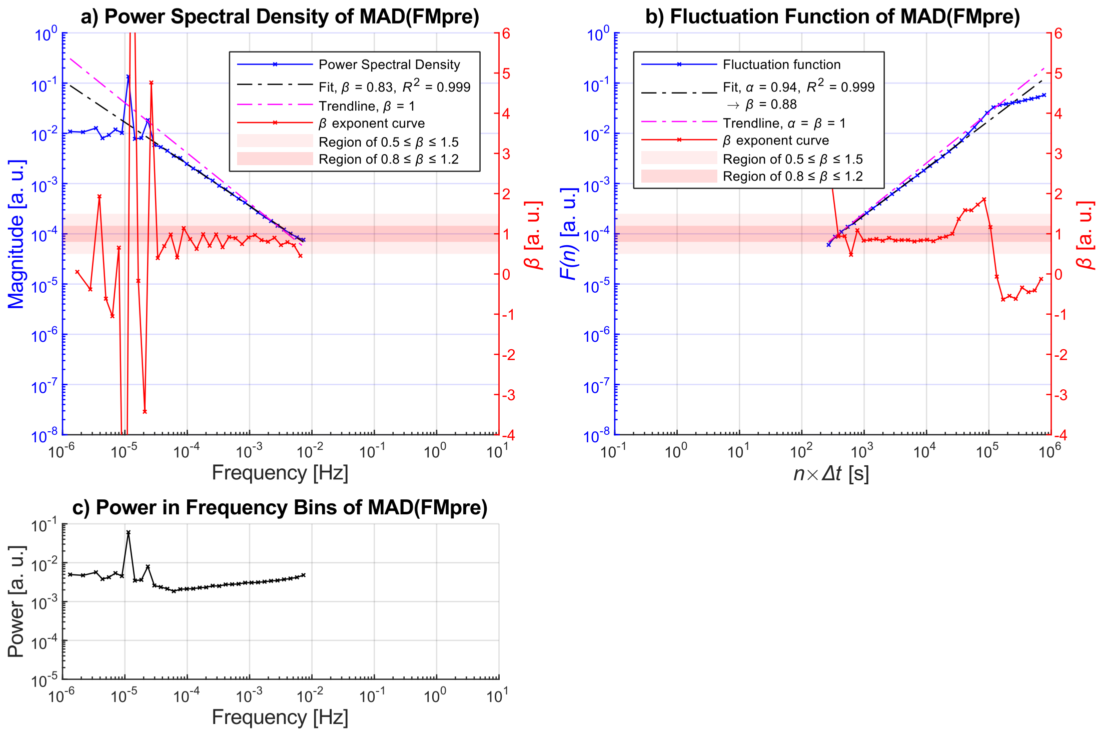


**Supplementary Figure S21. Ensemble-averaging-based results for the MAD(FMpre) activity signals.** For the description of the markings, see the caption of Supplementary Fig. S2.

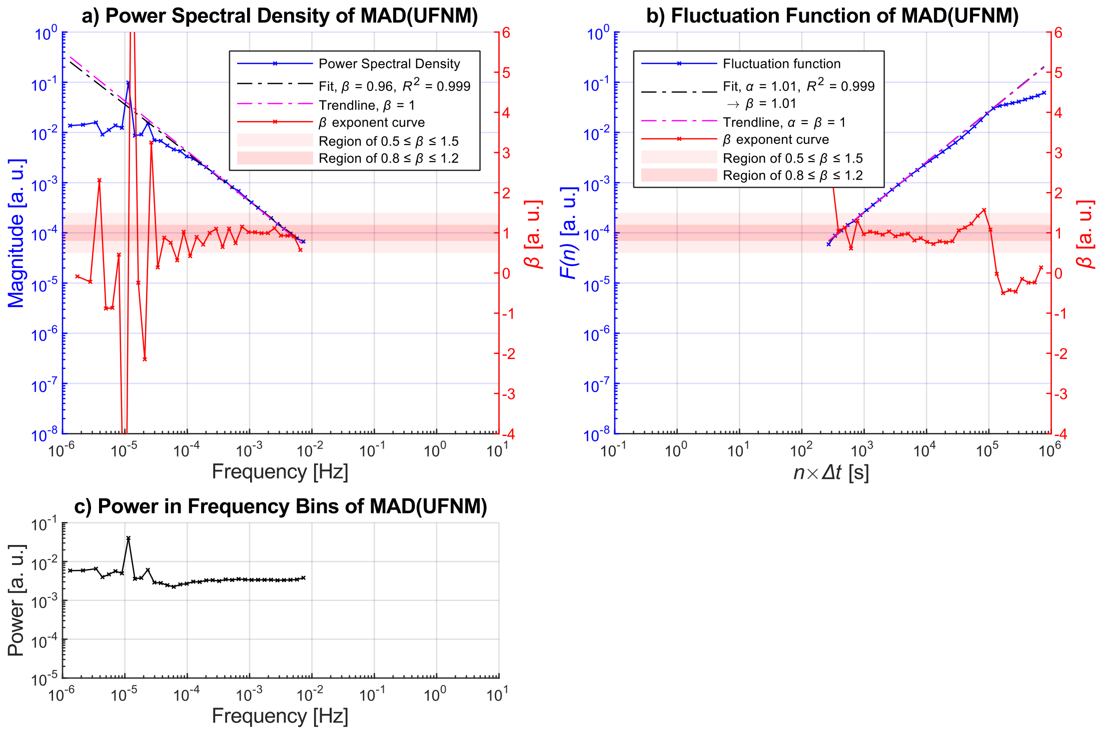


**Supplementary Figure S22. Ensemble-averaging-based results for the MAD(UFNM) activity signals.** For the description of the markings, see the caption of Supplementary Fig. S2.

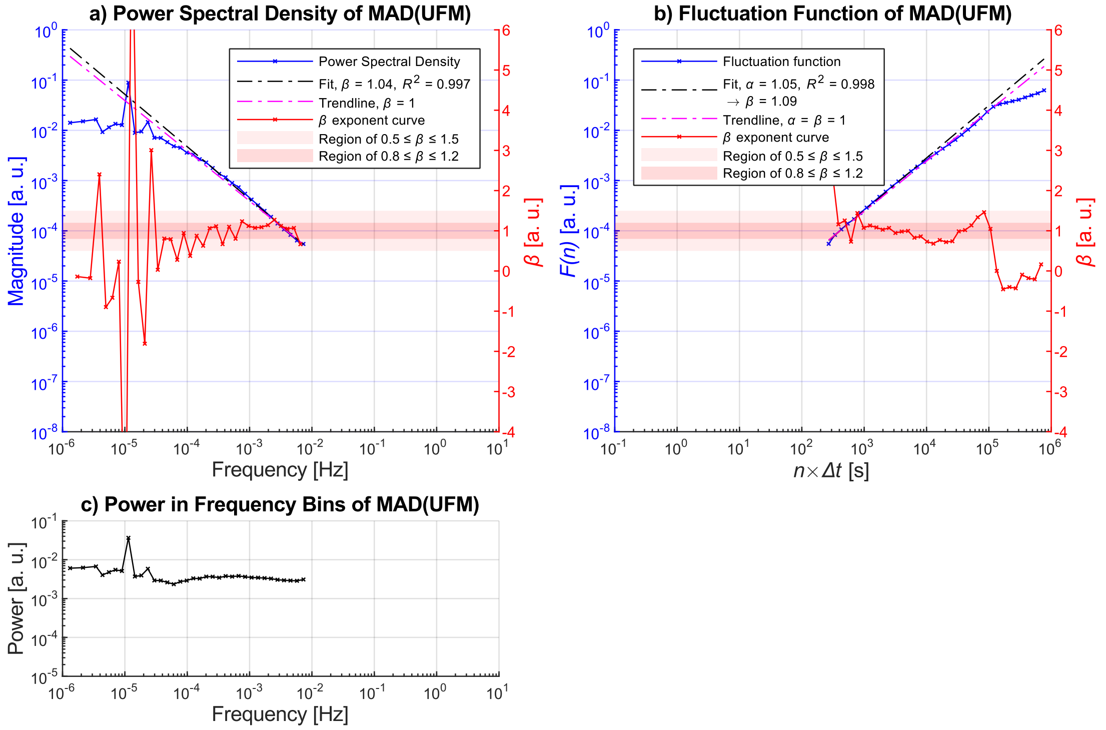


**Supplementary Figure S23. Ensemble-averaging-based results for the MAD(UFM) activity signals.** For the description of the markings, see the caption of Supplementary Fig. S2.

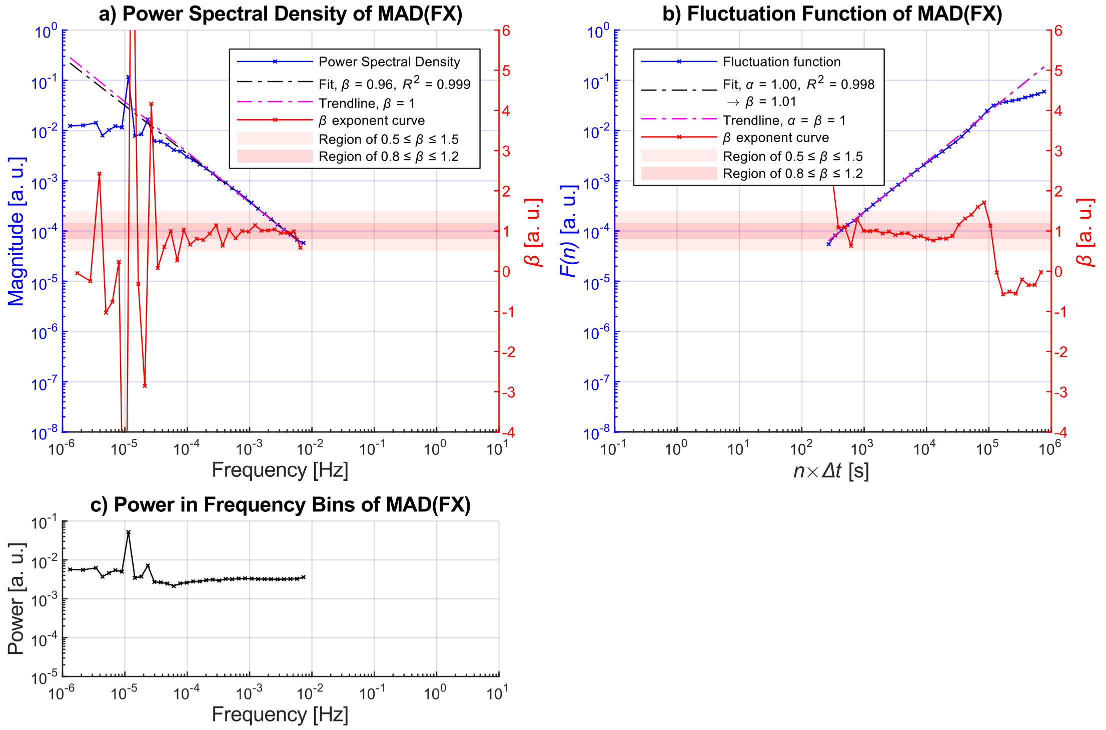


**Supplementary Figure S24. Ensemble-averaging-based results for the MAD(FX) activity signals.** For the description of the markings, see the caption of Supplementary Fig. S2.

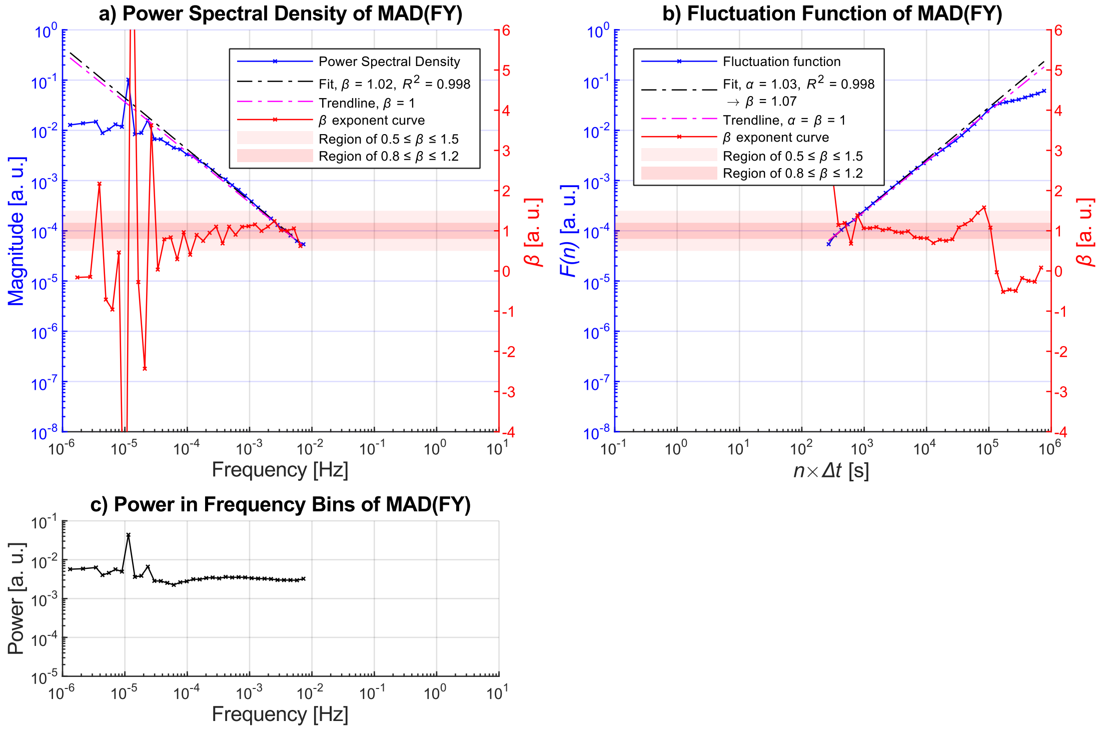


**Supplementary Figure S25. Ensemble-averaging-based results for the MAD(FY) activity signals.** For the description of the markings, see the caption of Supplementary Fig. S2.

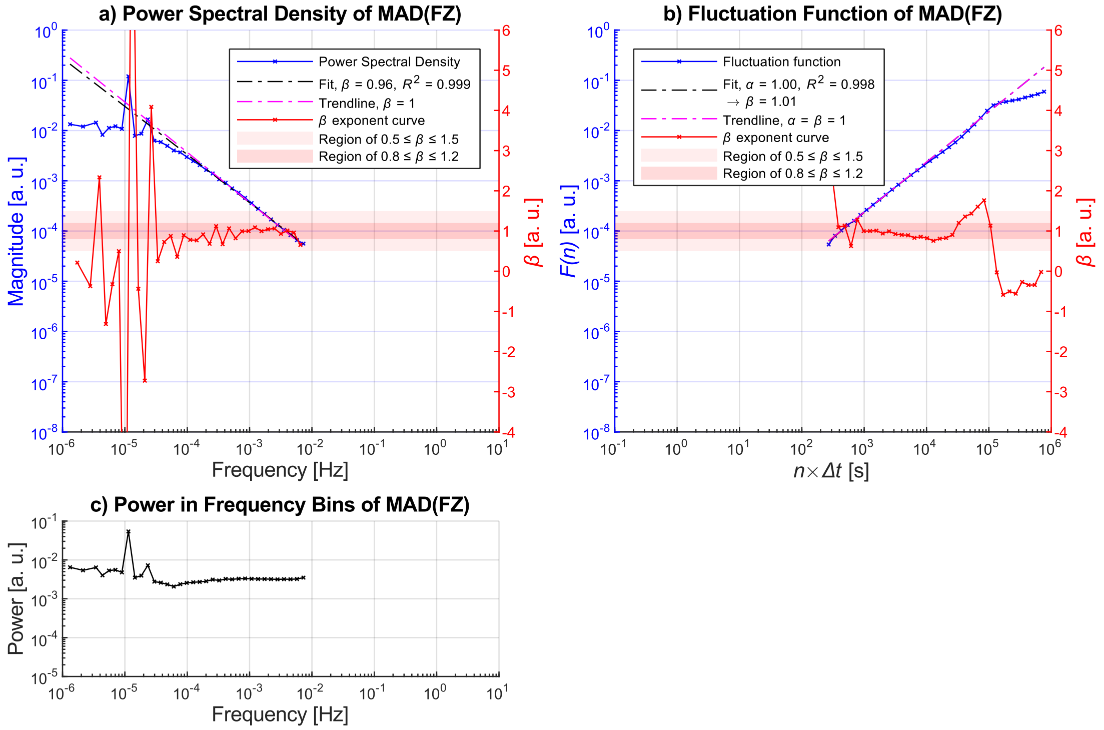


**Supplementary Figure S26. Ensemble-averaging-based results for the MAD(FZ) activity signals.** For the description of the markings, see the caption of Supplementary Fig. S2.

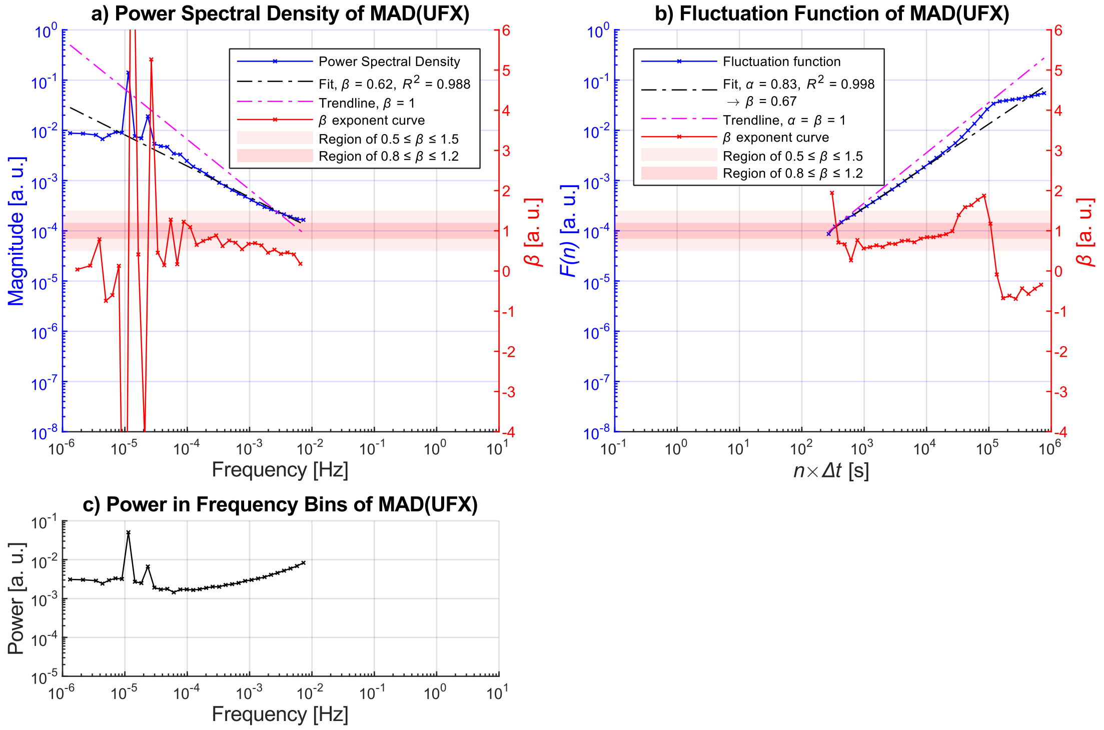


**Supplementary Figure S27. Ensemble-averaging-based results for the MAD(UFX) activity signals.** For the description of the markings, see the caption of Supplementary Fig. S2.

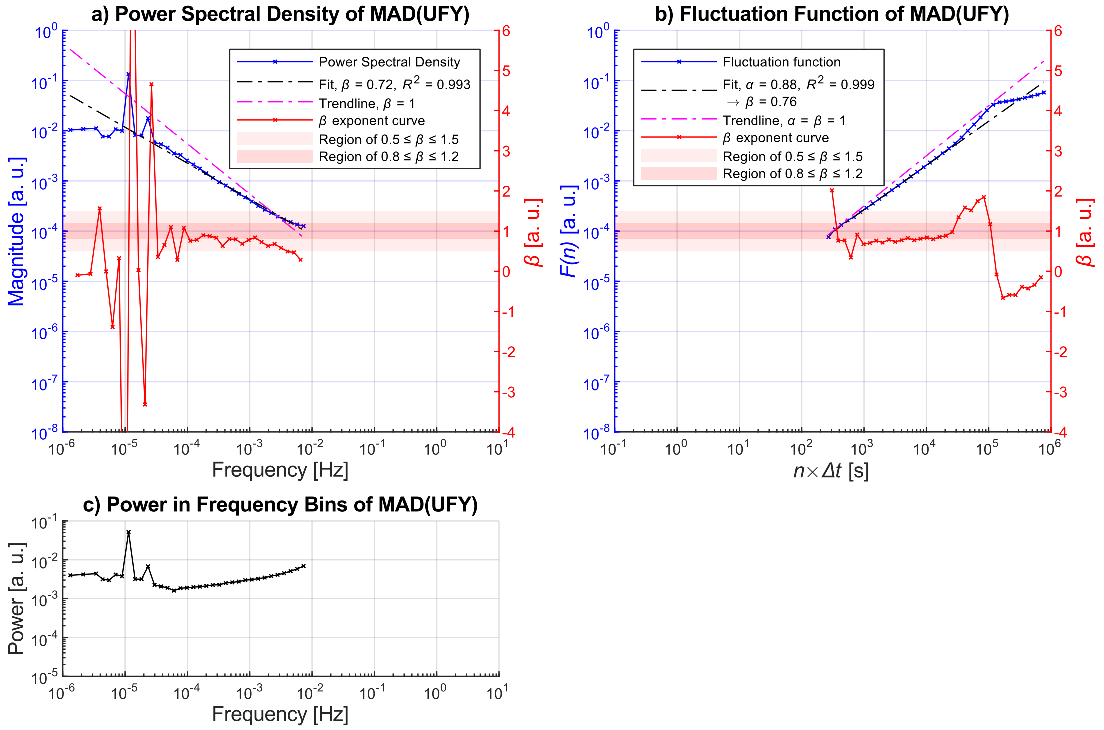


**Supplementary Figure S28. Ensemble-averaging-based results for the MAD(UFY) activity signals.** For the description of the markings, see the caption of Supplementary Fig. S2.

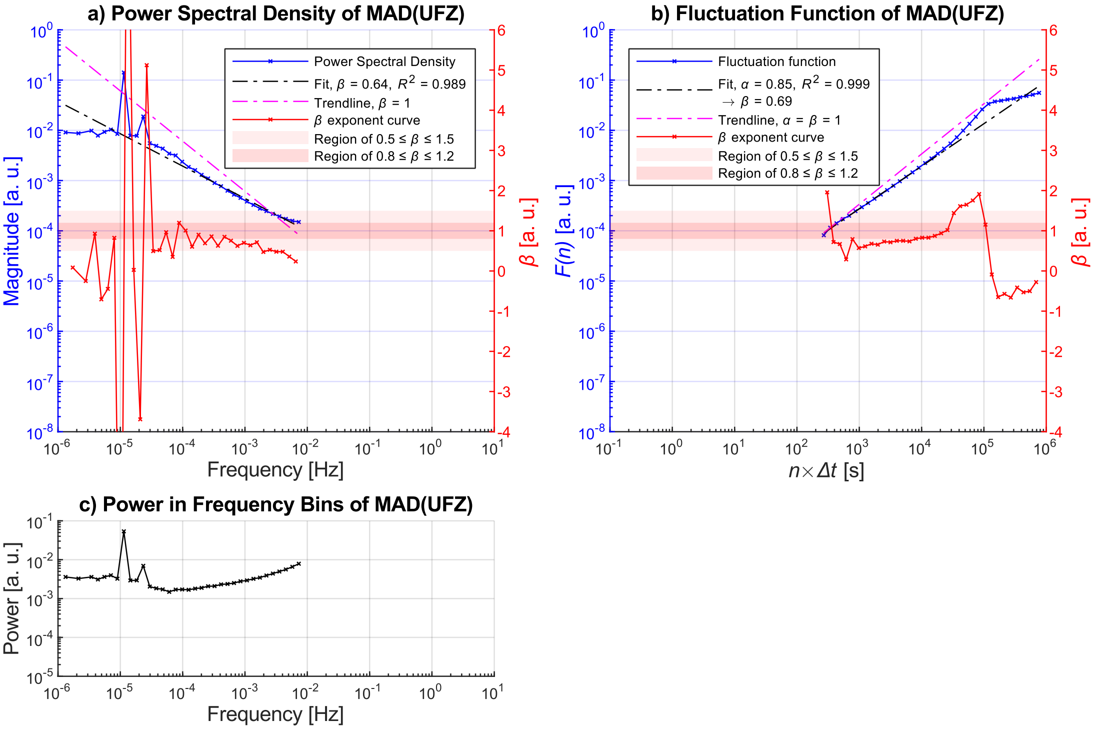


**Supplementary Figure S29. Ensemble-averaging-based results for the MAD(UFZ) activity signals.** For the description of the markings, see the caption of Supplementary Fig. S2.

## AI

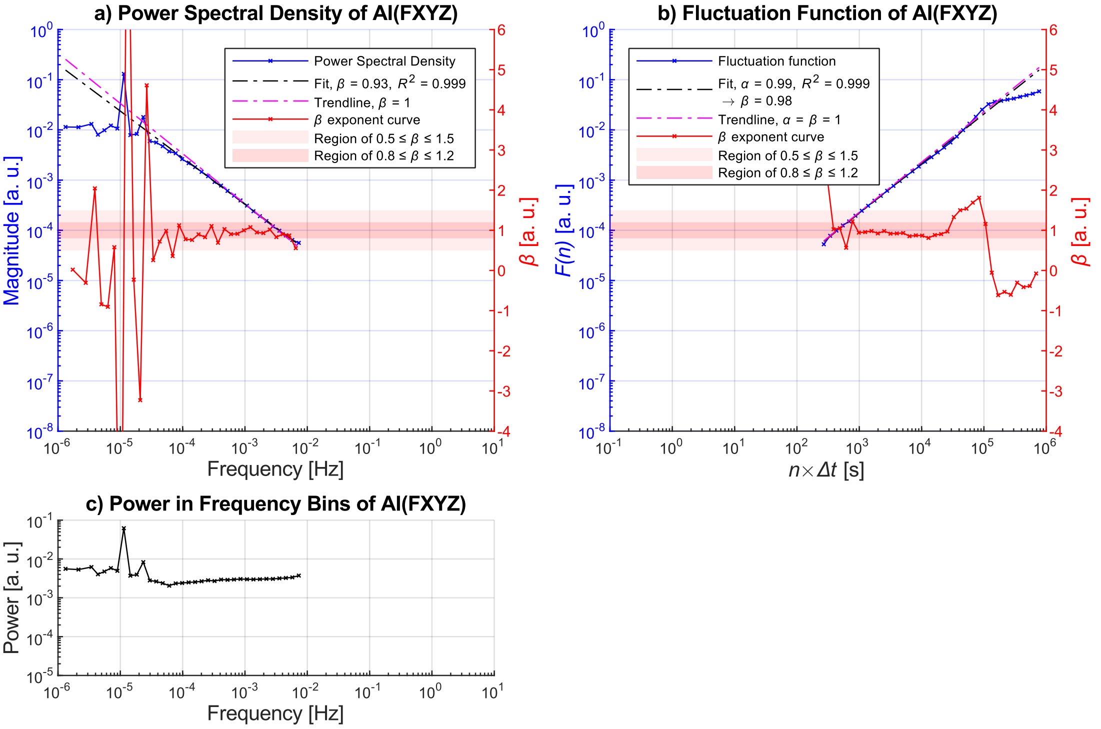


**Supplementary Figure S30. Ensemble-averaging-based results for the AI(FXYZ) activity signals.** For the description of the markings, see the caption of Supplementary Fig. S2.

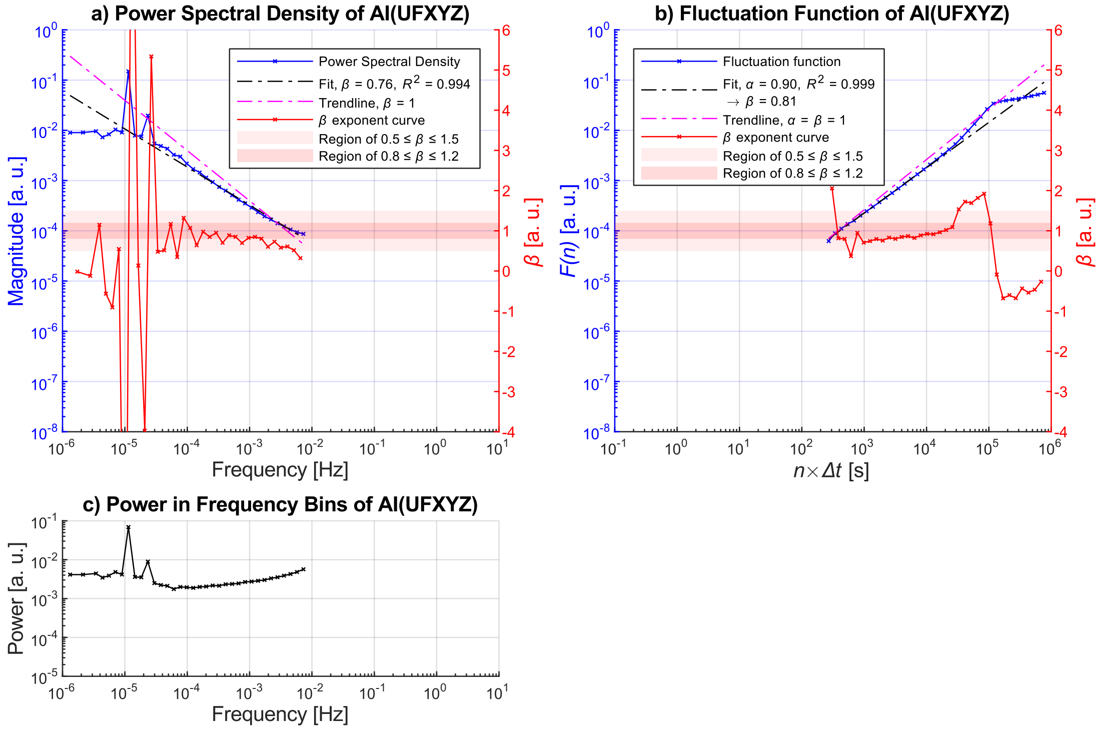


**Supplementary Figure S31. Ensemble-averaging-based results for the AI(UFXYZ) activity signals.** For the description of the markings, see the caption of Supplementary Fig. S2.

## ENMO

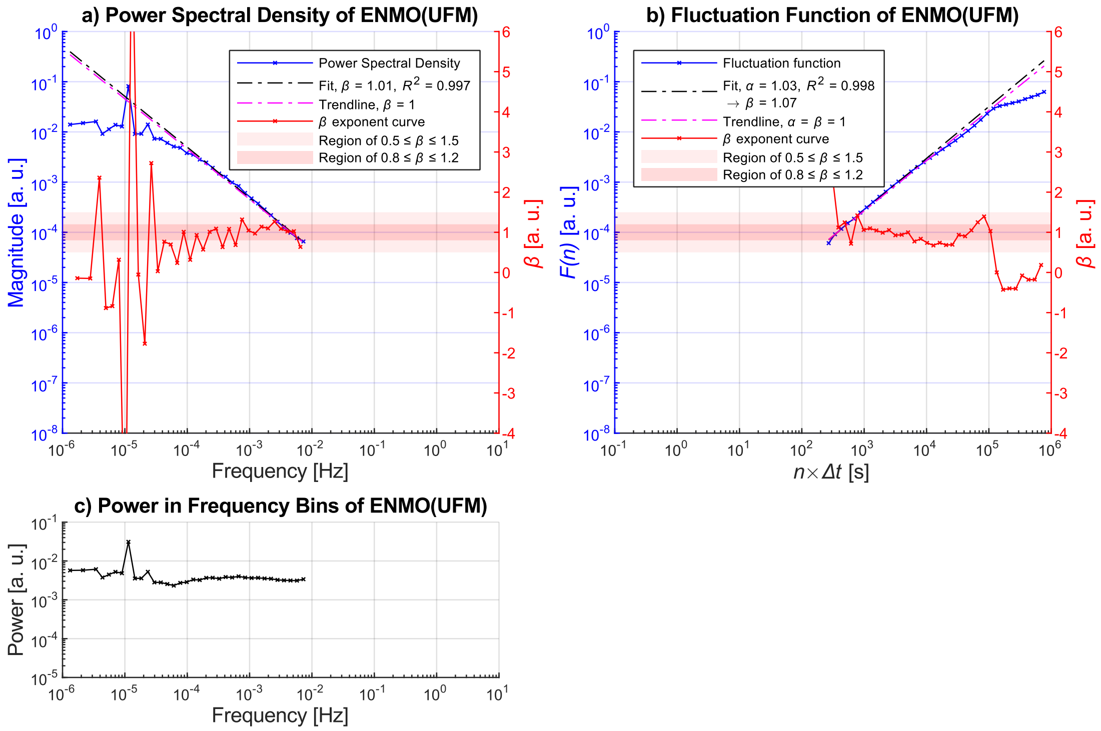


**Supplementary Figure S32. Ensemble-averaging-based results for the ENMO(UFM) activity signals.** For the description of the markings, see the caption of Supplementary Fig. S2.

## HFEN

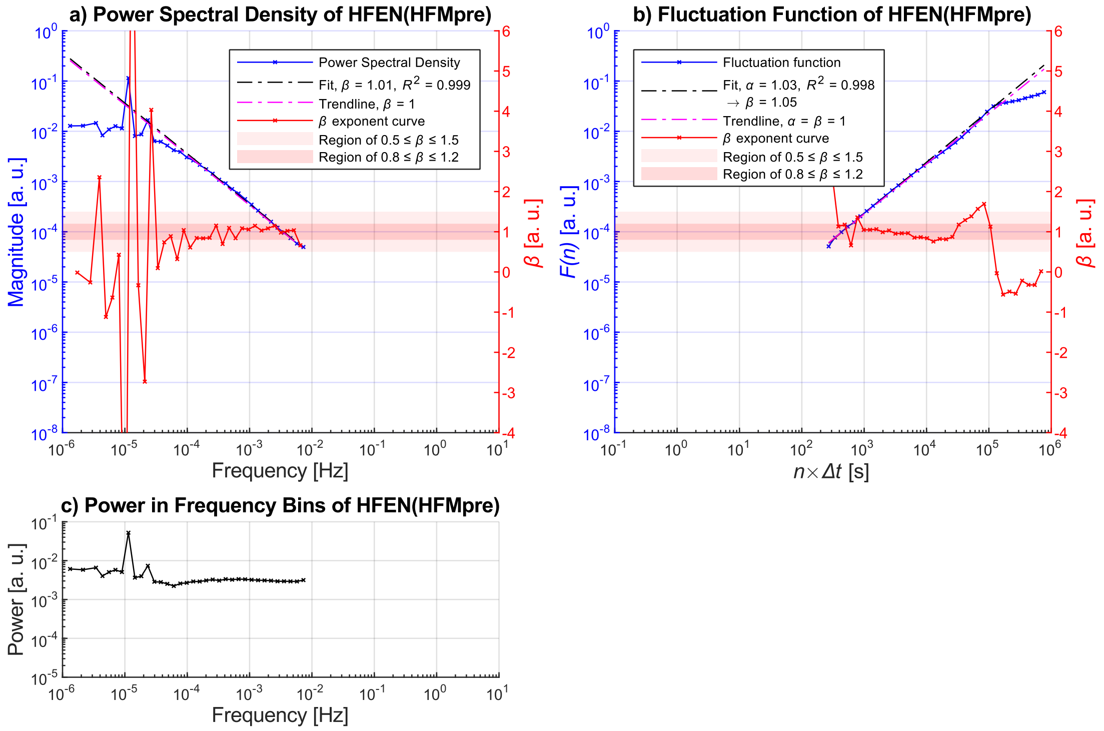


**Supplementary Figure S33. Ensemble-averaging-based results for the HFEN(HFMpre) activity signals.** For the description of the markings, see the caption of Supplementary Fig. S2.

## *Acceleration signals*

## HFMpre

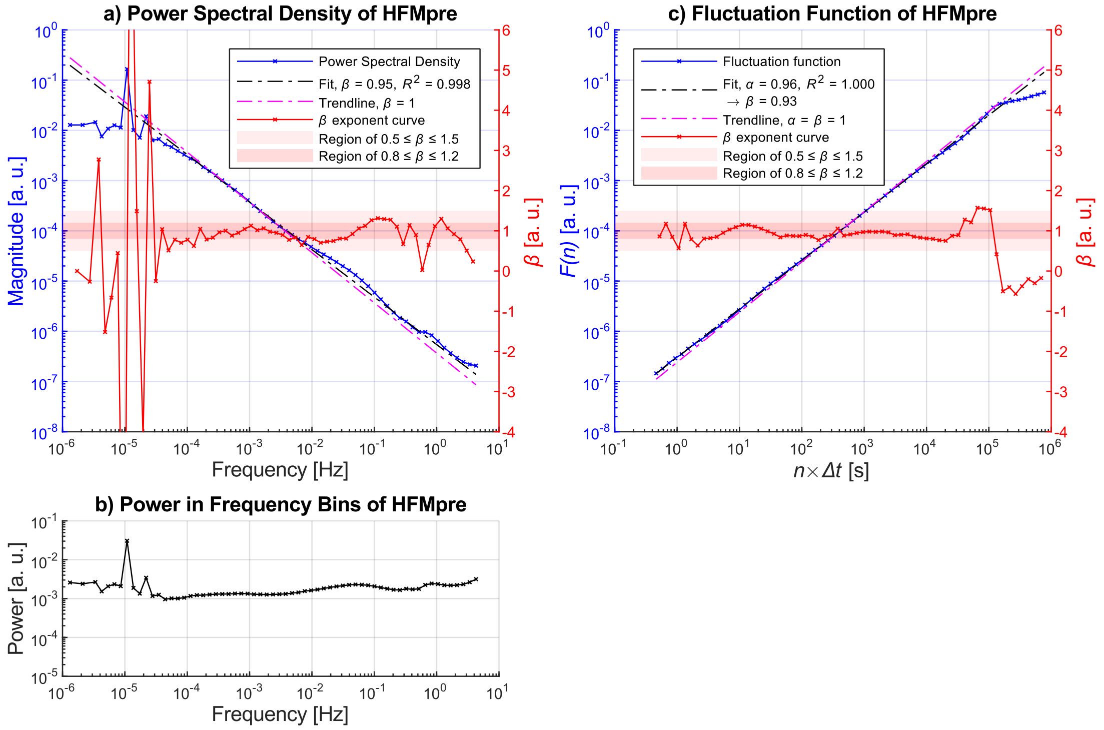


**Supplementary Figure S34. Ensemble-averaging-based results for the HFMpre acceleration signals.** For the description of the markings, see the caption of Supplementary Fig. S2.

## UFNM

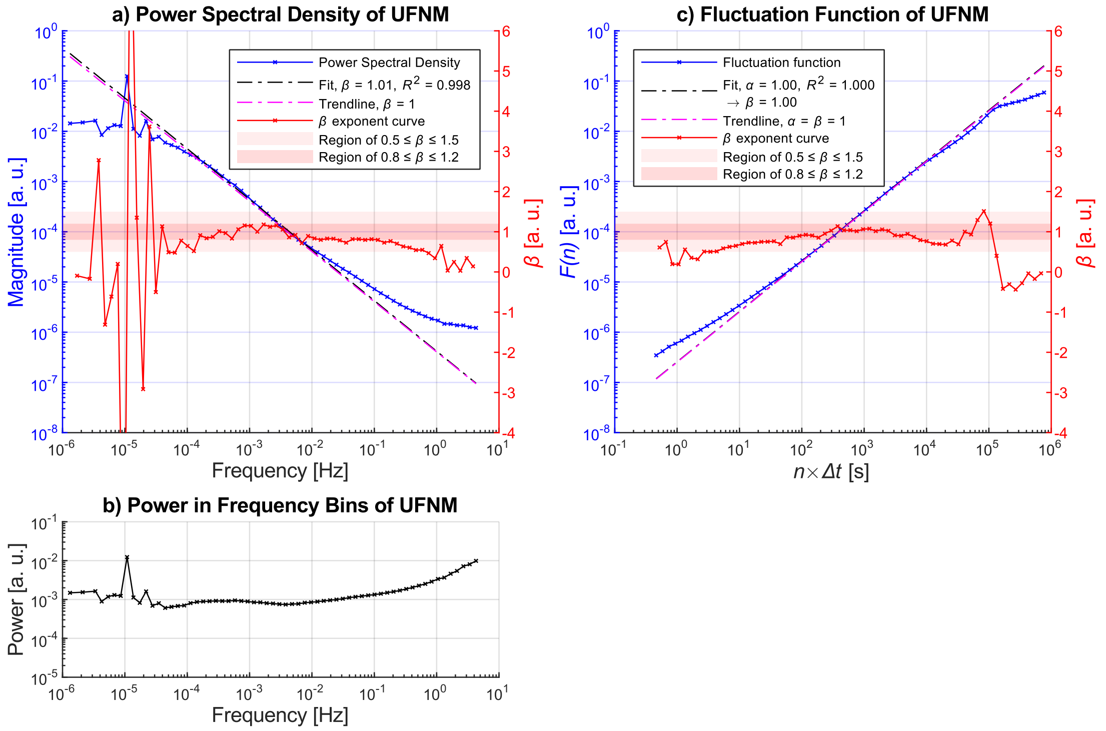


**Supplementary Figure S35. Ensemble-averaging-based results for the UFNM acceleration signals.** For the description of the markings, see the caption of Supplementary Fig. S2.

## UFX

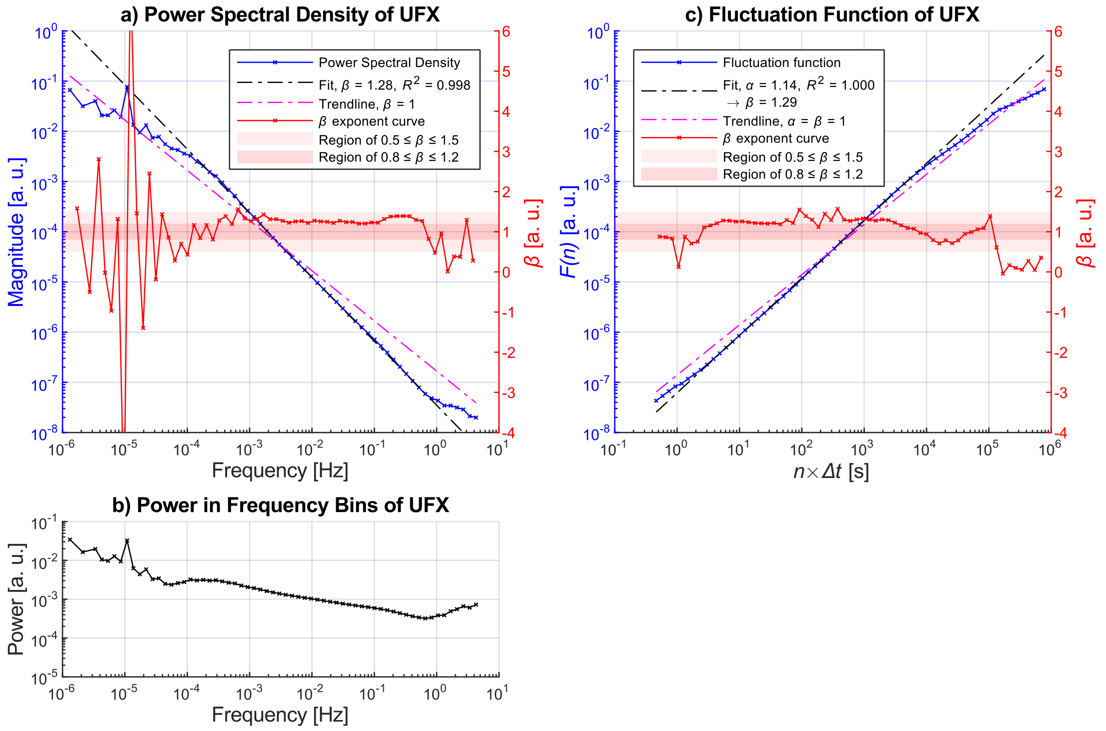


**Supplementary Figure S36. Ensemble-averaging-based results for the UFX acceleration signals.** For the description of the markings, see the caption of Supplementary Fig. S2.

UFZ

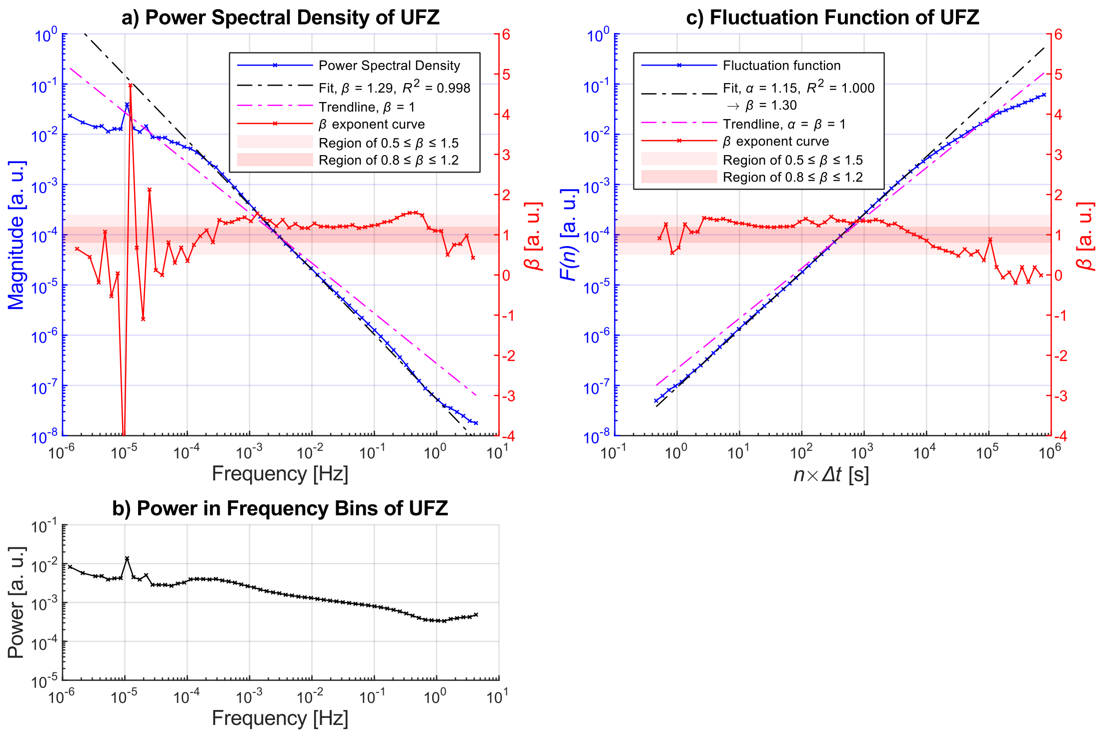


**Supplementary Figure S37. Ensemble-averaging-based results for the UFZ acceleration signals.** For the description of the markings, see the caption of Supplementary Fig. S2.

# References


[1] Bianchi S. fathon: A Python package for a fast computation of detrendend fluctuation analysis and related algorithms. Journal of Open Source Software 2020;5:1828. https://doi.org/10.21105/joss.01828.

[2] Gao L, Li P, Gaba A, Musiek E, Ju Y, Hu K. Fractal motor activity regulation and sex differences in preclinical Alzheimer’s disease pathology. Alzheimer s & Dementia Diagnosis Assessment & Disease Monitoring 2021;13. https://doi.org/10.1002/dad2.12211.

[3] Li P, Lim A, Gao L, Hu C, yu L, Bennett D, et al. More random motor activity fluctuations predict incident frailty, disability, and mortality. Science Translational Medicine 2019;11:eaax1977. https://doi.org/10.1126/scitranslmed.aax1977.

[4] Wohlfahrt P, Kantelhardt J, Zinkhan M, Schumann AY, Penzel T, Fietze I, et al. Transitions in effective scaling behavior of accelerometric time series across sleep and wake. EPL (Europhysics Letters) 2013;103:68002. https://doi.org/10.1209/0295-5075/103/68002.
